# Comparative evaluation of potential indicators and temporal sampling protocols for monitoring genetic erosion

**DOI:** 10.1111/eva.12197

**Published:** 2014-08-15

**Authors:** Sean Hoban, Jan A Arntzen, Michael W Bruford, José A Godoy, A Rus Hoelzel, Gernot Segelbacher, Carles Vilà, Giorgio Bertorelle

**Affiliations:** 1 National Institute for Mathematical and Biological Synthesis (NIMBioS), University of TennesseeKnoxville, TN, USA; 2 Department of Life Science, Università di FerraraFerrara, Italy; 3 Naturalis Biodiversity CenterLeiden, the Netherlands; 4 School of Biosciences, Cardiff UniversityCardiff, Wales, UK; 5 Estación Biológica de Doñana (EBD-CSIC)Seville, Spain; 6 Durham UniversityDurham, UK; 7 University FreiburgFreiburg, Germany

**Keywords:** alpha diversity, conservation biology, convention on biological diversity, microsatellites, population genetic, simulation, single nucleotide polymorphism, temporal sampling

## Abstract

Genetic biodiversity contributes to individual fitness, species' evolutionary potential, and ecosystem stability. Temporal monitoring of the genetic status and trends of wild populations' genetic diversity can provide vital data to inform policy decisions and management actions. However, there is a lack of knowledge regarding which genetic metrics, temporal sampling protocols, and genetic markers are sufficiently sensitive and robust, on conservation-relevant timescales. Here, we tested six genetic metrics and various sampling protocols (number and arrangement of temporal samples) for monitoring genetic erosion following demographic decline. To do so, we utilized individual-based simulations featuring an array of different initial population sizes, types and severity of demographic decline, and DNA markers [single nucleotide polymorphisms (SNPs) and microsatellites] as well as decline followed by recovery. Number of alleles markedly outperformed other indicators across all situations. The type and severity of demographic decline strongly affected power, while the number and arrangement of temporal samples had small effect. Sampling 50 individuals at as few as two time points with 20 microsatellites performed well (good power), and could detect genetic erosion while 80–90% of diversity remained. This sampling and genotyping effort should often be affordable. Power increased substantially with more samples or markers, and we observe that power of 2500 SNPs was nearly equivalent to 250 microsatellites, a result of theoretical and practical interest. Our results suggest high potential for using historic collections in monitoring programs, and demonstrate the need to monitor genetic as well as other levels of biodiversity.

## Introduction

A major tool in conservation biology is the temporal monitoring of biodiversity with indicators such as the number of species or size of populations. Monitoring indicators across time periods can often identify negative trends, with the ultimate goal being the detection of a decrease early enough to signal a conservation need (Noss 1990; Namkoong et al. 1996; Pereira and Cooper 2006). For example, Butchart et al. (2010) used temporal analysis of 31 indicators to evaluate the Convention on Biological Diversity (CBD)'s goal ‘to achieve a significant reduction of the current rate of biodiversity loss by 2010’. They showed convincingly that the goal was not met, as most indicators of pressure on biodiversity (e.g., harvest) increased while ‘state of biodiversity’ indicators (e.g., extent of habitat) declined. In evaluating the failure to meet the 2010 CBD goal, some have noted that indicators were either vague or developed too late, and that some indicators are less sensitive than others (Jones et al. 2011; Perrings et al. 2010; Nicholson et al. 2012). Thus, a current objective of several organizations (including the CBD and the Intergovernmental Panel on Biodiversity and Ecosystem Services, IPBES) is to develop appropriate indicators for comparing recent, current and future biodiversity for species, ecosystems and services, as well as genetic diversity (Jones et al. 2011; Hoban et al. 2013a; Pereira et al. 2013).

Genetic diversity was previously neglected in official biodiversity policy (Laikre et al. 2010) but has recently received more attention (Sgrò et al. 2011; Santamaria and Mèndez 2012; Hoban et al. 2013a). Target 13 of the new CBD Strategic Plan (https://www.cbd.int/sp/) aims to ‘minimize genetic erosion’ and ‘safeguard genetic diversity’ in species of agricultural, socio-economic and cultural importance. National and international resource management agencies, especially forestry, fisheries, and agriculture, are also seeking to monitor and preserve genetic diversity of the wild populations they utilize (Brown 2008; FAO 2010; Pinsky and Palumbi 2014). There is therefore an urgent need for policy-relevant studies to help define sensitive and robust indicators of genetic diversity change, as well as appropriate genetic sampling protocols, knowledge that is currently lacking (Schwartz et al. 2007; Brown 2008; Hoban et al. 2013a; Pereira et al. 2013).

The value of genetic diversity is increasingly recognized for contributing to individual fitness, species' evolutionary potential, and ecosystem function and resilience (Hughes and Stachowicz 2004; Reusch et al. 2005; Whitham et al. 2008). It is also recognized that genetic diversity loss increases species' vulnerability, lowers fitness, and accelerates the path to extinction (Spielman et al. 2004; Frankham 2005). Thus, governmental and commercial entities are increasingly attentive to the need to monitor the genetic status and trends of wild populations' genetic diversity, to inform policy decisions and management actions (e.g., protected areas, harvest limits, restoration). Measures of variation, what is termed ‘evolutionary currency’ (Parenti 1982), are basic and relevant quantities that may be of prime interest to monitoring projects. Many conservation studies over recent decades have empirically measured genetic parameters such as allelic diversity or heterozygosity over time (Nielsen et al. 1997; Spencer et al. 2000; Vilà et al. 2003; Zhu et al. 2013), and these types of studies are increasing due to technical advances allowing genetic analysis of low quality/quantity DNA, for example, using non-invasive and historical samples (Farrington and Petren 2011; Casas-Marce et al. 2012). Such temporal studies typically incorporate different numbers of time points, individual samples and genetic markers, and temporal intervals between sampling. However, the power to detect ongoing genetic erosion using various simple and direct measures of genetic diversity, with different sampling schemes has not been quantified. Quantitative advice on temporal monitoring methodologies is thus urgently needed to optimize efforts of conservation researchers and practitioners.

Our goal was to determine what genetic metrics are most sensitive and robust, what temporal sampling protocols are appropriate, and what genetic markers show sufficient resolution, on conservation-relevant time scales. Here, we use realistic, individual-based simulations to evaluate the power of six potential indicators and various sampling designs to detect genetic erosion after demographic decline. The evaluated indicators, which are common summaries of a population's genetic status (Allendorf and Luikart 2007) are number of alleles, allelic size range, observed heterozygosity, expected heterozygosity, the Garza-Williamson *M*-ratio bottleneck statistic, and Wright's inbreeding coefficient (*F*_is_). These indicators are here tested under different population decline models, sampling efforts, and sampling schemes. Recognizing that many species of concern now have sufficient genomic resources available, we also test the power of moderate and large numbers of loci. We answer the following questions: (i) which indicator is most sensitive to the decline, (ii) how many temporal samples are needed, (iii) what temporal sampling pattern (interval between time points) is most appropriate, (iv) is power of an indicator dependent on the type of population decline, and (v) what is the effect of sample size and marker number on power? We also test the ability to detect genetic change after population demographic recovery. We evaluate two types of genetic markers: microsatellites, which are currently the most common markers in ecology and conservation and for which baseline data are available from many endangered species, and single nucleotide polymorphisms (SNPs), which are rapidly emerging as an affordable, high-throughput, high genome-coverage marker.

## Methods

### Simulation

Simulated or synthetic data created under known conditions can be used to evaluate performance of analytical methods. Such evaluations help inform the proper use of the methods in applied settings. Simulation approaches such as repeated-sampling and coalescent methods have been widely used in population genetics and conservation biology for decades. More recently, individual-based simulations have gained popularity by incorporating greater realism, which enables more thorough assessment of how a method can be expected to perform in real-world conditions (Landguth et al. 2010; Hoban et al. 2012; Hoban 2014). We therefore used the simulation software Nemo (Guillaume and Rougemont 2006) to perform generation-by-generation, individual-based simulations of populations that experience realistic demographic decreases. We then sampled from the simulated data sets and analyzed the samples using six indicators. In this way, we could compare the relative performance of each indicator under known conditions.

In our simulations, individuals are male or female, mate at random, and produce Poisson distribution of offspring (mean = 2). We simulated a single population with census size *N* = 2000 (previously run 10 000 generations to reach equilibrium, see Figure S1) undergoing exponential decline for three primary situations: decline to *N* = 200, *N* = 50, and *N* = 20 (90%, 97.5%, and 99% decline). We designate these (relatively) as weak, moderate, and strong declines. Decline occurs over 10 generations, thus a 97.5% exponential decline gives population sizes at each generation of: 2000, 1383, 956, 661, 457, 316, 219, 151, 105, 72, and 50 (Figure S2). Note that forward-in-time individual-based simulations, including Nemo, generally model (by their individual-based nature) census population size *N*_c_. Of course, effective population size (*N*_e_) is the parameter directly influencing loss of genetic diversity (Antao et al. 2011; Frankham et al. 2014). Considering that the model in Nemo has Poisson distributed contributions from each parent, and all individuals are semelparous, our individual-based model will be close to *N*_c_/*N*_e_ = 1, though this situation may be uncommon in nature. Researchers should consider our simulation results as reflective of changes in a species whose *N*_c_ approximates *N*_e_. Note also that Nemo sets absolute carrying capacity and that the per generation *N*_c_ is usually slightly (approximately 1–10%) below this (Table S1). We first simulated exponential decline to represent gradual habitat loss or climate-induced decline, which is the most common shape exhibited by animal population declines (Di Fonzo et al. 2013). However, we previously showed that bottleneck signatures may differ for instant and gradual size changes (Hoban et al. 2013b). As such, it is possible that power to monitor genetic diversity loss may also differ for these two models of population decrease. Therefore, we additionally performed simulations for instant decline (in one generation), of the same degree as above, for example, 97.5% instant decline gives population sizes at each generation of: 2000, 50, 50, 50 … 50. Instant declines may occur from disease outbreak, natural or anthropogenic catastrophe (e.g., oil spill, volcano eruption), population collapse (e.g., fisheries), or sudden over-exploitation of wildlife resources (e.g., American bison, northern elephant seal, exotic pet trade).

To determine if results depend on initial population size we performed additional simulations from initial *N* = 10 000 to *N* = 300, 100, and 50 (97%, 99%, and 99.5% declines). Here, the 99% decline allows direct comparison of magnitude to the 99% situation described above, while the decline to 50 allows direct comparison to final population size of 50 described above. Additionally, we simulated populations that instantly recover (more precisely, carrying capacity is instantly raised to its original value; census *N* takes several generations to fully recover after this) to original size after periods of 2, 10, and 20 generations of low population size to determine whether increases in genetic metrics are detectable. We performed 100 replicates of every scenario (a complete list of scenarios is presented in Table S2). Each replicate was run for 10 000 generations to reach equilibrium, so each simulation replicate has an independent starting condition.

### Sampling

In general, 50 samples were taken every generation and genotyped at 20 microsatellite DNA loci. In the 99% decline only 20 individuals remained and all were sampled. Twenty microsatellites provide a level of resolution that is consistent with many ongoing studies. We also tested additional sampling approaches: all individuals in the population (thus no sampling error), and 50 samples but 250 microsatellites. For two scenarios (moderate instant and moderate exponential decline), we tested also a scheme of 50 samples at 2500 SNPs.

We calculated number of alleles (*K*), allelic size range for microsatellites (*K*_r_), observed and expected heterozygosity (*H*_o_, *H*_e_), the Garza-Williamson *M*-ratio bottleneck statistic for microsatellites (GW), and Wright's inbreeding coefficient (*F*_is_). An important caution for real monitoring programs is that if sample sizes are not equal for all time points sampled, and sampling is not exhaustive, allelic richness determined by rarefaction should be substituted for number of alleles, *K* (see Discussion). Sampling and analysis were performed with the custom-made software ConvFstat (available at sites.google.com/site/hoban3/scripts), which samples, converts file formats and runs arlecore, a command-line version of Arlequin (Excoffier and Lischer 2010). We then performed two statistical tests (described below) on these indicators, using (R Core Development Team, 2013), to estimate power for detecting significant temporal changes in these potentially informative indicators.

### *T*-tests

Samples from each generation were compared pairwise to samples from every other generation. Paired tests, with loci as replicates, have been used previously to compare modern and historical samples (Schwartz et al. 2007; Dornelas et al. 2013; Pinsky and Palumbi 2014). The series of comparisons results in a half-matrix of *P*-values for whether the indicator value at each generation significantly differs from the indicator value computed at every other generation. We summed the number of significant tests (*P* < 0.05) over replicates, for each pair-wise comparison and each indicator. This sum, divided by 100 (the number of replicates), represents the power to conclude whether the genetic indicator is significantly different between any two generations.

We note that samples from consecutive generations are non-independent, and that in an empirical study testing a series of null hypotheses, a multiple comparisons correction would be warranted. However, our simulation study is constructed to mimic a real monitoring program in which a monitor would be making a single or very small number of pairwise comparisons. For example, a real monitor might choose to sample at *T* = 1 and *T* = 8 and would therefore perform one statistical test; the single null hypothesis would be that a diversity metric does not differ between the two time points. We aimed in our study to report the power that a real investigator could likely expect in this situation, and thus multiple comparison correction would not be appropriate for our work. Our results will help inform a future monitoring program about which intergenerational comparisons could be effective, and thus when to sample.

### Anova

We tested 20 specific temporal sampling schemes, for example, different combinations of generations to be sampled. These schemes vary in terms of different number of samples, different temporal ‘clumping’ of samples, and whether or not samples are available before decline begins (Table1, Figure S2). For example, one scheme is to sample at generations 1, 3, 5, 7, 9, and 11. For each replicate, we performed repeated-measures anova to test whether the factor ‘time of sampling’ significantly explains variation in the genetic indicator, with variation across loci encompassed in an error term. Simple anova and repeated-measures anova have been used in previous genetic diversity temporal studies (Reusch et al. 2005; Farrington and Petren 2011). The test results in a *P*-value, and (using a threshold of *P* < 0.05) a conclusion as to whether a significant difference was observed. Summed over replicates and divided by 100, this represents power of a given sampling scheme to conclude that genetic diversity is declining over time in a given situation.

**Table 1:** Descriptions of 20 monitoring schemes- generations to be sampled. Population decline occurs after generation one.

Description of sampling scheme	Generations sampled
Every generation sampled	1-2-3-4-5-6-7-8-9-10-11
Even spread, six samples, including one before decline	1-3-5-7-9-11
Even spread, six samples, but none available before decline	3-4-6-8-9-11
Clustered, six samples at beginning and end	1-2-3-9-10-11
Clustered, six samples at beginning and end, but none available before decline	3-4-5-9-10-11
Clustered, six most recent generations	6-7-8-9-10-11
Even spread, four samples, including one before decline	1-5-8-11
Even spread, four samples, but none available before decline	3-5-8-11
Clustered, four samples at beginning and end	1-2-10-11
Clustered, four samples at beginning and end, but none available before decline	3-4-10-11
Only four recent samples	8-9-10-11
Clustered, four samples at beginning and end, but most recent two not available	1-2-8-9
Clustered, four samples at beginning and end, but first two and most recent two not available	3-4-8-9
First and most recent generation	1-11
Early and most recent	3-11
Middle and most recent	6-11
Late and most recent	8-11
First and middle	1-6
First and late	1-8
First and penultimate	1-10

## Results

**Figure 1:**
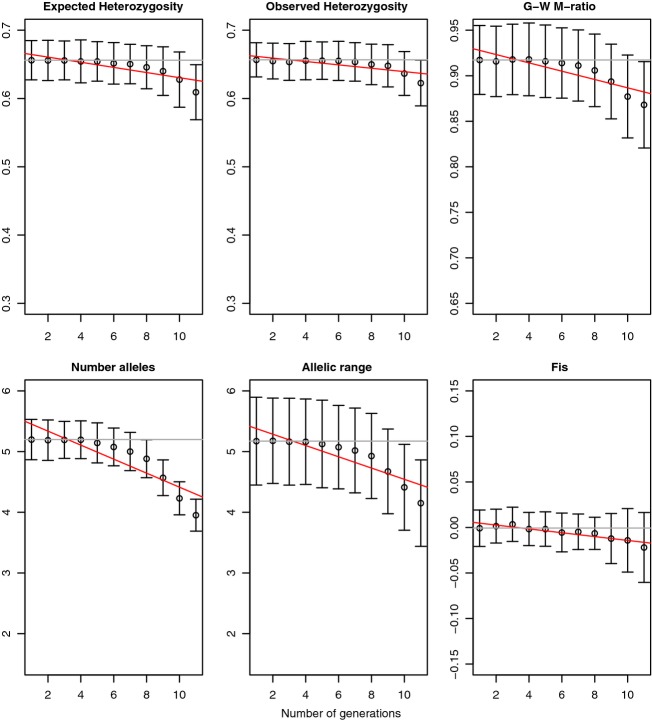
Genetic response (*y*-axis) to 99% *exponential* population decline, measured at 20 loci, in 50 individuals, over generations (*x*-axis). Error bars represent standard deviation. Linear regression of the indicator is shown with a red line. Gray line is value at generation one.

**Figure 2:**
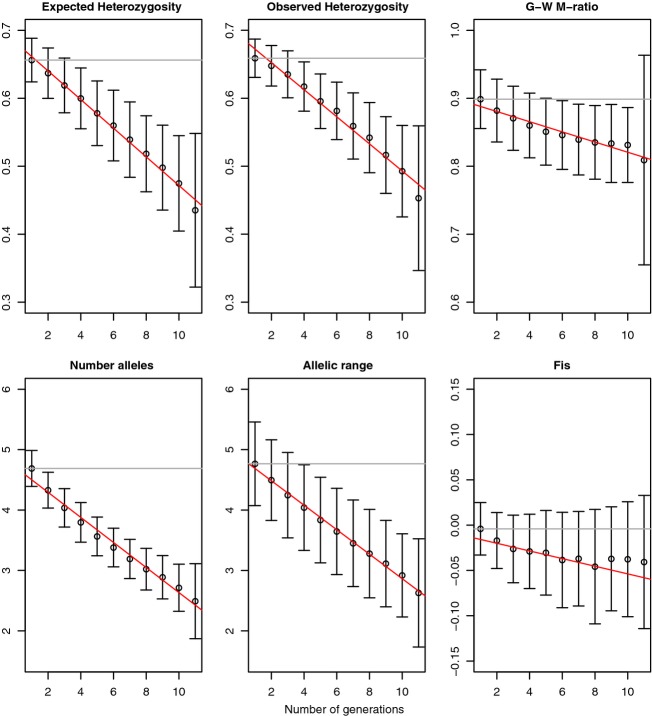
Genetic response (*y*-axis) to 99% *instant* population decline, measured at 20 loci, in 50 individuals, over generations (*x*-axis). Error bars represent standard deviation. Linear regression of the indicator is shown with a red line. Gray line is value at generation one.

The simulated populations showed realistic numbers of alleles (mean 5.21 for *N* = 2000, 11.62 for *N* = 10 000) and heterozygosity (0.66 for *N* = 2000, 0.85 for *N* = 10 000). Theoretical expectations for heterozygosity for a population of *N*_e_ = 2000 is 0.667 (Hedrick 2011), and for number of alleles is 4.81 under the Kimura and Ohta (1975) approximation. Expected number of alleles for *N*_e_ = 10 000 is approximately 10.5 based on coalescent simulations (Hoban et al. 2013c). As expected, genetic indicators generally decreased following population decline in all situations (Figs1 and 2), but the degree of genetic loss, the time lag, and the ability to detect it, varied among the indicators tested and among the types of decline.

### *t*-Tests results

#### Exponential decline

**Figure 3:**
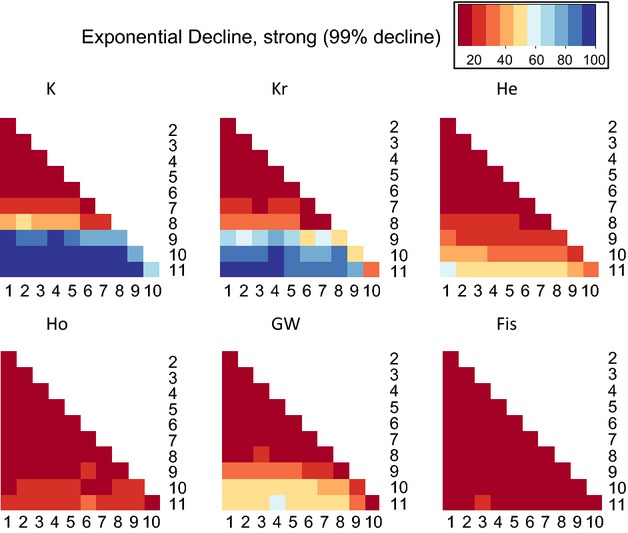
Pairwise comparisons showing the proportion of 100 replicates (i.e., power) in which the indicator at generation X was significantly different from the indicator at generation Y, for the scenario strong (99%) exponential decline from *N* = 2000. Darkest blue is power >0.90. Power <0.50 is orange shades, and power <0.10 is dark red. Abbreviations as in Table2.

Overall, *K* (and to a lesser degree *K*_r_) shows high power and outperformed the other indicators, which typically show <0.50 power, often much less (Fig.3, Data S1). In the case of strong (99%) decline, using *K*, the power for detecting significant differences was substantial (0.70 or higher) for comparing the three most recent generations (when populations size is lowest) to previous generations. In the case of moderate (97.5%) decline, substantial power was obtained only when comparing the most recent generation to previous generations; all other inter-generational comparisons showed low power. For weak (90%) decline, power never exceeds 0.20 for any indicator for any intergenerational comparison.

#### Instant decline

**Figure 4:**
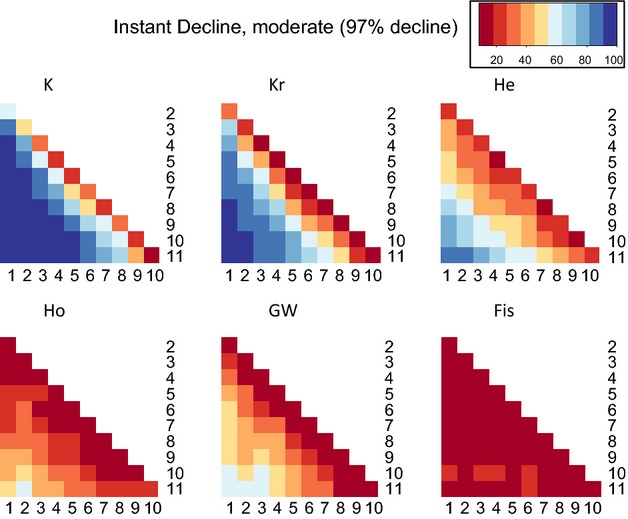
Pairwise comparisons showing the proportion of 100 replicates (i.e., power) in which the indicator at generation X was significantly different from the indicator at generation Y, for the scenario moderate (97%) instant decline from *N* = 2000. Darkest blue is power >0.90. Power <0.50 is orange shades, and power <0.10 is dark red. Abbreviations as in Table2.

Overall, power was much higher for instant than for exponential declines (Fig.4, Data S1). Importantly, a response became evident more swiftly, in as few as one or two generations, for strong and moderate cases. This is readily observed in the half-matrix of power for instant declines which shows power increasing steadily with time between samples, whether early or late in the bottleneck, making the matrix relatively symmetric (though with some loss of power in the latest generations, see Discussion). In contrast the matrix is highly asymmetric for exponential declines (Fig.3). For most pairwise comparisons, especially for comparisons separated by two or more generations, using *K*, power for instant declines was >0.90. On the other hand, a response was still nearly undetectable for weak cases, with power reaching >0.50 only after eight generations and never exceeding 0.62. Again, *K* and *K*_r_ perform best, but other indicators (especially *H*_e_) performed well in some cases (Figs2 and 4, Data S1).

### anova results

#### Exponential decline

As with t-tests, the indicators *K* and *K*_r_ showed highest utility (Table2, Data S1). Monitoring using six samples provided higher power than four, which provided higher power than two; sampling all 11 generations provided the highest power (Table2). However, assuming the final generation (generation 11, lowest population size) was sampled, the difference in using 2, 4, 6, or 11 samples was minor. For example, there was little difference in power between schemes sampling generations 1-2-10-11 and 1-11. Furthermore, if the two most recent generations were unsampled (when the population is smallest and subject to the greatest genetic loss), power was low (consistent with *t*-test results). For example, sampling generations 1-2-8-9 (for moderate exponential decline, for *K*), had <0.30 power to detect differences, while sampling generations 1-2-10-11 had power of 0.80 (Table2). Additionally, temporal arrangement (clumped or evenly spread, pre-decline sample availability, etc.) did not substantially affect power (e.g., 1-5-8-11 vs 1-2-10-11). Thus, important factors were time between samples (e.g., 1-11 was better than 3-11), time since start of the decline, and whether samples were available from periods of small population size (e.g., 6-11 was better than 1-6).

**Table 2:** Number of significant anovas for each monitoring scheme over 100 replicates, for each indicator (columns), for exponential (top section) and instant decline (bottom section). Moderate (97.5%) decline is shown as representative, full results in Data S1.

Sampling scheme	*H*_o_	*H*_e_	*K*	*K*_r_	GW	*F*_is_
Exponential						
1-2-3-4-5-6-7-8-9-10-11	3	27	79	57	21	9
1-3-5-7-9-11	4	20	81	57	23	6
3-4-6-8-9-11	5	19	80	52	23	9
1-2-3-9-10-11	3	24	79	59	24	6
3-4-5-9-10-11	6	24	78	59	26	7
6-7-8-9-10-11	2	18	73	51	20	8
1-5-8-11	3	25	81	54	23	9
3-5-8-11	7	20	79	54	23	6
1-2-10-11	1	26	80	57	23	10
3-4-10-11	6	22	79	60	22	9
8-9-10-11	3	16	68	43	19	7
1-2-8-9	1	14	27	25	3	8
3-4-8-9	3	7	14	15	4	8
1-11	2	16	79	50	22	13
3-11	8	15	77	51	16	10
6-11	7	17	67	47	17	11
8-11	6	13	59	40	17	10
1-6	4	6	8	7	2	6
1-8	5	6	18	12	2	4
1-10	5	13	51	33	14	4
Instant						
1-2-3-4-5-6-7-8-9-10-11	57	93	100	100	72	12
1-3-5-7-9-11	50	88	100	100	70	9
3-4-6-8-9-11	43	75	100	94	63	8
1-2-3-9-10-11	64	93	100	100	72	14
3-4-5-9-10-11	47	83	100	97	62	13
6-7-8-9-10-11	24	64	90	77	37	9
1-5-8-11	44	86	100	100	66	12
3-5-8-11	40	73	100	94	55	10
1-2-10-11	65	91	100	100	71	9
3-4-10-11	49	79	100	95	65	12
8-9-10-11	14	38	76	49	19	8
1-11	45	84	100	99	60	10
1-2-8-9	39	76	100	99	65	5
3-4-8-9	30	59	99	89	56	6
3-11	40	73	100	89	51	8
6-11	26	58	90	67	29	15
8-11	15	37	66	43	9	7
1-6	18	47	100	88	43	8
1-8	28	66	100	92	50	10
1-10	35	80	100	98	57	11

*H*_o_, Observed heterozygosity; *H*_e_, expected heterozygosity; *K*, number alleles; *K*_r_, allelic range; GW, Garza-Williamson *M*-ratio statistic; *F*_is_, inbreeding coefficient.

#### Instant decline

Again, power was much higher under instant than exponential decline (Table2). Similar to the results above, more temporal sampling points yielded higher power, though if the first and final generations were included, the gain in power from including more time points was minor. In marked contrast to results for the exponential decline, the temporal arrangement of samples following instant decline (clustered samples, before and after start of decline, etc.) did substantially affect power. Specifically, when we compared sampling schemes that were identical except for whether or not a sample was obtained before the decline (i.e., the first sample at generation 3 instead of generation 1), we found that the unavailability of samples prior to decline typically resulted in approximately 0.20 lower power, depending on the indicator. Temporally clustered samples (two at the beginning and two at the end) yielded slightly higher power than regular sampling (equidistant temporally). Also in marked contrast to exponential declines, the unavailability of the most recent generations did not reduce power, for example, schemes 1-6 and 6-11 were similar. (Actually, sampling 1-6 performed slightly better – see Discussion regarding power reduction for the later generations). It should be noted that, for *K*, most sampling schemes perform reasonably well for moderate and strong declines (power often >0.90), and poorly for weak ones (power typically <0.70).

### Other simulations

In all additional simulations the strong effects of decline type and weaker effects of particular sampling strategy were apparent, and the best indicator remained *K* (Figure S3). Unsurprisingly, greater power was achieved for a decline from *N* = 10 000 to 50 than from *N* = 2000 to 50. When considering a decline of equivalent percentage, the 97% and 99% declines from *N* = 10 000 showed less power than the 97% and 99% declines from *N* = 2000.

**Figure 5:**
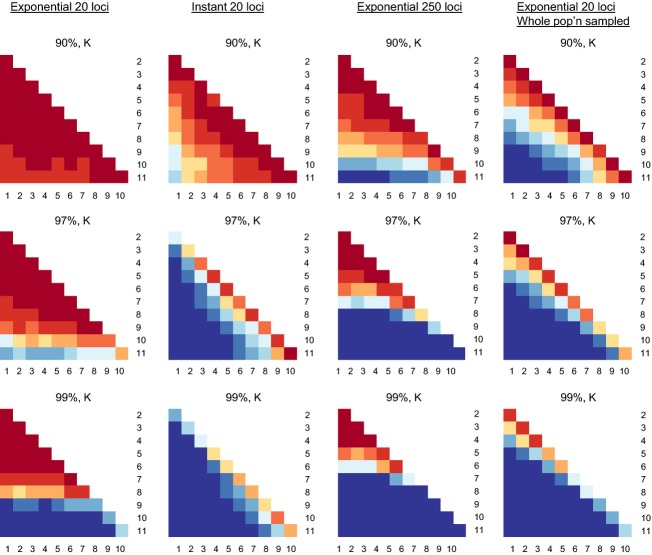
Comparison of the proportion of 100 replicates (i.e., power) in which the indicator at generation X was significantly different from the indicator at generation Y, contrasting population scenarios that vary the kind of population decline, number of microsatellite markers analyzed and sample size (columns) with results for *K*; otherwise as Fig.3. Top, middle, and bottom panels show results for weak (90%), moderate (97%), and strong (99%) decline. Abbreviations as in Table2 (pop'n-population).

As expected, genotyping 250 microsatellites achieved higher power than 20. Nonetheless, there was still low power early in the exponential decline – reasonable power was not achieved until seven generations after decline or later (Fig.5). Genotyping 2500 SNPs achieved approximately similar (though slightly higher) to 250 microsatellites (Figure S4). Note that approximately half the SNPs are monomorphic at equilibrium.

Sampling the entire population represents the maximum obtainable power for a given number of markers. Under this condition, power is substantially increased over a sample size of 50, especially for moderate and weak bottlenecks (Fig.5).

**Figure 6:**
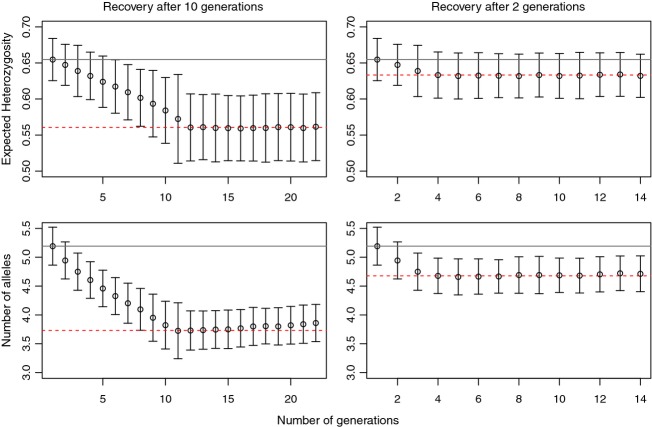
Number of alleles and heterozygosity, through time, under a scenario in which near-instantaneous population recovery takes places either 10 or two generations after a period of a small size due to instant decline. Indicator values before the decline and after the recovery are shown by gray solid and red dashed lines, respectively.

In spite of a slight upward response in indicators following full demographic recovery, there was essentially no power to detect genetic diversity increase, for all three recovery situations, for any indicator (Figure S5). Nonetheless, we observed that recovery after two generations resulted in a population that lost approximately one-third of the heterozygosity and number of alleles that would be lost in a more delayed recovery (5% heterozygosity loss and 10% allelic loss for a two generation decline, vs 15% and 30% loss after 20 generations, Fig.6). Thus, genetic erosion can be halted quickly via demographic recovery, although the genetic erosion that has occurred is essentially irreversible on small time-scales (tens of generations) and genetic indicators will not substantially increase (noted also in Nei 1975).

## Discussion

Consistent with population genetic theory predicting that alleles are lost rapidly during population size reductions (Nei et al. 1975) and empirical observations in small populations (Spencer et al. 2000), the number of alleles (*K*) showed the clearest response and highest power for monitoring genetic decline across all scenarios (Figs4). Larsson et al. (2008) also observed more rapid and more significant response in *K* than heterozygosity (*H*_e_) in simulations parameterized to black grouse, as did Pinsky and Palumbi (2014) in simulations of fish stock declines. While the strong performance of *K* might have been predictable *a priori* from a general and qualitative standpoint, our work is the first quantitative and direct comparison of the utility of six genetic diversity metrics for genetic monitoring following a wide variety of realistic scenarios of population decline (see Carvajal-Rodríguez et al. 2005 for an evaluation of the utility of quantitative traits, though on a longer time scale of 100 generations). We show that, with microsatellites, measuring *K* is often 2–4 times more powerful than using *H*_e_ (Table2), and *K* is the only metric that consistently yields power >0.90 across many situations. Moreover and perhaps most importantly we show that power differed strongly for type and severity of decline, but less strongly for most sampling schemes.

Principally, we showed that power critically depends on the type and severity of demographic decline. All genetic indicators show markedly lower power under exponential population decline (Figs4, Table2). For this decline type, loss of genetic diversity is difficult to identify by temporal monitoring: (i) during the initial generations of even severe population size declines (i.e., there seems to be low potential for these indicators as very ‘early warning’ signs), (ii) if samples are not available from periods of low population size, for example, *N* < 100, or (iii) if samples are extremely close in time. This is consistent with theoretical expectations that population size, time between samples, and time since start of demographic decline will affect genetic erosion. Under instant decline, on the other hand, using *K*, decreased genetic diversity can be identified with good power within one or two generations after decline, for severe (99%) or moderate (97.5%) declines, and notably even while population size remains constant. However, weak (90%) declines yielded lower power, in agreement with Pinsky and Palumbi (2014), who tested power to detect genetic erosion after 90% instant declines in large fish stocks. Our results suggest that genetic erosion may not be detectable with these indicators in some cases of substantial, ecologically-relevant demographic decline. One example would be taxa that are deemed ‘Critically endangered’ under International Union for the Conservation of Nature (IUCN) criteria after ≥80% population size reduction over the last three generations (http://www.iucnredlist.org/technical-documents/categories-and-criteria/2001-categories-criteria). Our results should not be interpreted that weak declines are unimportant, only that the resulting genetic loss may be difficult to detect in the short term.

Our results also indicate several considerations regarding sampling scheme during temporal genetic monitoring. In both types of decline (exponential and instant), results show that as few as two temporal samples can reveal genetic diversity loss (with *K*) when declines were moderate or severe and when sampling spans the period of decline. For exponential declines, power is largely unaffected by the spacing of samples in time, and whether two, four, six, or more samples are taken. For instant declines, power is usually improved by approximately 0.20 if samples are available before the onset of the decline as compared to when sampling started multiple generations into the decline. This result emphasizes the importance of ancient or archived/museum samples (Larsson et al. 2008; Magurran et al. 2010; Jackson et al. 2011). On the other hand, clustered sampling is only slightly better than regular sampling. Indeed, many schemes were sufficient, and strict adherence to a particular sampling protocol seems unnecessary. Under exponential or gradual declines it seems never ‘too late’ to initiate a monitoring program, in particular if some historical material is available (even if collected for another reason, e.g., museum specimens). However, for instant declines, some later pairwise comparisons (e.g., comparing generations 8 and 10) show low or no power (Fig.4), possibly because alleles at many loci become fixed, after which no significant differences could be further observed. Therefore, if historical samples are unavailable, for instant declines in particular, monitoring schemes should be implemented as soon as possible, before all variation disappears. Samples taken for monitoring should be stored securely for future analysis with new genetic techniques that may arise (Schwartz et al. 2007; Magurran et al. 2010).

With reasonable numbers of markers and samples, once a population is small (*N *∼ 100), genetic diversity loss should be readily identifiable before severe erosion occurs. Specifically, by examining the mean values of *K* at each generation (e.g., Figs1 and 2, exact values not shown), we observed that approximately 85–90% of alleles remain when the signal of loss is first detected under exponential severe declines (at generation 9), under exponential moderate decline (at generation 11), under instant moderate decline (at generation 4), or under instant severe (at generation 3). Percentages are lower (70–80%) if diversity loss is calculated on the entire population rather than the sample, as noted by Pinsky and Palumbi (2014), showing that many alleles lost are extremely rare. In any case, we recommend using *K* for genetic biodiversity monitoring as feasible and affordable for a range of population decline situations. However, it must be noted that *K* is very sensitive to sample size (Allendorf and Luikart 2007), so monitors should strive for equal sample sizes. When sample sizes differ, investigators must adjust the value of *K* using a technique such as rarefaction (e.g., allelic richness).

As expected, power increased by increasing the number of markers. However, under the exponential decline model, even 250 microsatellites or 2500 SNPs rarely yielded good power in the initial generations. Sampling all individuals provided a clearer signal (Fig.5), but still did not succeed in the earliest stages of exponential decline. While sampling the entire population is more powerful than using many loci, such sampling scheme may be impossible, so it is important to note that increasing the number of loci also increased power. Microsatellites are commonly used in genetic studies, but new methods, including restriction site associated DNA sequencing, now permit screening thousands of loci at comparable costs. (Notable, developing a SNP chip may be cost effective for repeat screening over time.) Four of the six indicators (excepting allelic size range and *M*-ratio) that we evaluated can be calculated for any previously popular (e.g., allozymes) or future (e.g., SNPs or genome sequencing) marker types. Individual SNP loci are generally less polymorphic than microsatellite loci. In our study, 2500 SNPs were comparable to 250 microsatellites, suggesting that ability to detect genetic losses may depend on the total number of alleles available. One issue with SNPs and microsatellites is ascertainment bias- during marker development, loci are usually chosen to be polymorphic in the current population, thus genetic losses may be underestimated. Our simulations did not replicate such bias. Monitoring adaptive genetic biodiversity is also emerging as a complement to neutral markers (Brown 2008; Hansen et al. 2012). It would be worthwhile for a future evaluation study to test indicators on adaptive-linked loci, because diversity at adaptive markers may be more resilient under population decline (Aguilar et al. 2004), depending on the type of locus-specific selection in operation. It may also be worth evaluating the utility of metrics specifically applied to SNP markers.

When population decline was exponentially convex, the response of most genetic diversity metrics was exponentially concave over time (slow then rapid loss), and when population decline was instantaneous the genetic response was linear (Figs1 and 2, Figure S4). This phenomenon is also reflected in the near-symmetry of the heat matrix for instantaneous decline. Larsson et al. (2008) also observed near-linear loss in *K* after near-instantaneous population collapse in black grouse. This has some implications for the general shape of genetic response that can be expected relative to the shape of population decline- almost certainly the curves will not be of the same shape, and genetic erosion will not be proportional to population loss (see also Nei 1975). Another important observation is that genetic diversity loss does accumulate over time even after the population contraction has stopped, and can be quite rapid in small constant-size populations. Additionally, the standard deviation of most metrics increases moderately through time (on the time scale we tested; standard deviations should diminish once new equilibrium is reached). This means that forecasts of the exact genetic diversity impact on a population may inherently exhibit increasing uncertainty through time, making it difficult to predict exact genetic composition into the future.

New variation is only introduced via mutation in an isolated population (significant increase of genetic variation can occur via migration), so once diversity is lost, it is very slowly restored even if population size fully recovers. Thus, monitoring genetic increase after population size recovery has essentially no power. However, if population decline was recent, rapid population demographic recovery can instantly halt loss of remaining diversity, as illustrated in Fig.6 and in recent empirical work (Brekke et al. 2011). Facilitating population recovery as fast as possible is clearly crucial for demographic (Martin et al. 2012) and genetic reasons, and would constitute the best possible intervention for isolated populations. We emphasize, of course, that monitoring census size in many species, especially following size changes, will be a poor indicator of genetic loss, as the effective size may be orders of magnitude smaller than the census size especially during recovery from a small size. We also emphasize that monitoring effective size accurately is difficult, and that direct genetic metrics may be preferred.

In addition to showing differing sensitivity, the indicators we tested summarize different aspects of genetic diversity. Number of alleles and allelic range are analogous to alpha diversity in ecology (i.e., richness, or count data) while heterozygosity is a measure of evenness. In both disciplines, the two aspects are typically correlated. The most responsive indicator, allelic diversity, is notable because it directly represents extinction of genetic variants, with direct consequences for alleles with present or future functional/adaptive importance. The G-W *M*-ratio and *F*_is_ are ‘compound’ indicators that are often interpreted, when computed on a single time sample, as evidence of recent bottlenecks or excess homozygotes in small populations, respectively. Both estimators, perhaps due to their variance, perform poorly. It is unsurprising that *F*_is_ shows little response as our populations are modeled with random mating. It may be surprising that *M*-ratio performs more poorly than the statistics on which it is based (namely number of alleles and allele size range), though this statistic has high variance and moreover was designed to detect extremely severe declines. It is currently unclear how other ‘synthetic’ (a.k.a. ‘compound’, or ‘higher-level’) indicators perform relative to basic metrics such as number of alleles. If ‘compound’ statistics in general show high variance, direct and more easily interpretable genetic measures may be most promising. Work to develop further metrics is required. Possibilities include use of temporal *F*_ST_ or the allele frequency spectrum to monitor another aspect of genetic erosion (drift in allele frequencies); these could complement the indicators we investigated (Schwartz et al. 2007). Quantitative genetic variation is another possibility (Carvajal-Rodríguez et al. 2005). These are all ‘state’ indicators; work is also needed to test the utility of ‘pressure’ indicators such as degree of fragmentation or harvest, or domesticated-wild hybridization.

Our study focused on genetic erosion, but temporal monitoring may be desired for other reasons such as changes in genetic connectivity due to fragmentation or to monitor the response of a population to genetic restoration or ‘genetic rescue’, such as via translocation (Vilà et al. 2003; Landguth et al. 2010; Aitken and Whitlock 2013). It remains to be tested whether the sampling schemes and indicators that we tested could be appropriate for such goals. Our results do emphasize that stable but small populations (e.g., prairie chicken, Mauritius kestrel) that are often currently only monitored for population size should undergo genetic monitoring to evaluate the success of these programs. It should also be noted that ancient DNA samples are often scattered across time and therefore samples from several time points are sometimes combined (due to insufficient samples at each single time point) to represent one time period, which may be problematic for analyses. An alternative is to use individual-based metrics and methods (J. Godoy, unpublished data).

While our study highlights general points regarding the potential of genetic monitoring, we considered a limited set of conditions. Simulations can be used to tailor monitoring programs to particular species' life histories that are known to affect retention of genetic diversity, for example, overlapping generations, variance in reproductive success, sex ratios (Hoban et al. 2013c,d; Pinsky and Palumbi 2014), or to test other realistic conditions (e.g., linear population decline, uneven sample sizes). A single population was modeled in this study, representing an isolated population or the species as a whole. However, many organisms are organized as metapopulations, where genetic diversity may decline more slowly with increasing levels of connectivity (Pinsky and Palumbi 2014). Genetic differentiation among demes and current gene flow are parameters to monitor in such context.

Lastly, in order to avoid ‘describing the world's fate ever more precisely while doing nothing to avoid it’ (Fischer et al. 2012), monitoring programs should be connected to broader conservation policy (Martin et al. 2012; Nicholson et al. 2012; Lindenmayer et al. 2013) rather than simply being used as ‘record-keeping’. Specifically, indicators should provide a signal for action (e.g., active habitat protection and management, perhaps supplemented with captive breeding, translocation). Thresholds or ‘red flags’ for genetic diversity loss (e.g., 10% decline in *K*), and plans of appropriate actions to implement after thresholds are observed (Martin et al. 2012), require discussion and establishment by the population genetic community. Much further work is needed to incorporate genetic status and trends into policy (Brown 2008; Laikre et al. 2010; Santamaria and Mèndez 2012; Hoban et al. 2013a).

## Summary

The power to detect genetic erosion differed strikingly for exponential and instant declines, and among indicators, while the precise number and distribution of temporal samples available has less effect. The often limited effect of differing temporal distribution of samples shows that opportunistically collected museum or archival specimens can be utilized effectively in genetic monitoring. Typing a relatively small number of loci appears adequate and cost-efficient, especially under the most dangerous condition of rapid and severe population declines. Fortunately genetic diversity loss can be detected while the vast majority of original allelic diversity remains, in time to signal need for conservation actions.

Notably, we observe that substantial genetic diversity loss does not occur even 10 generations after a population is reduced from *N* = 2000 to *N* = 200, but becomes quickly detectable once *N *∼ 50 or 100. This threshold matches some proposed Minimum Viable Population Sizes and agrees with empirical observations of genetic loss (Allendorf and Luikart 2007; Larsson et al. 2008). We conclude that monitoring genetic erosion may be unfeasible and perhaps unnecessary when *N*_e_ (effective population size) exceeds several hundreds or the decline is very recent (a few generations), even when using many genetic markers. Monitoring will likely be most effective once population size is small (*N*_e_ < 100), and sampling should increase in frequency after this size threshold. Small but constant-size or slowly recovering populations are also in need of intense genetic monitoring. This concurs with Lindenmayer et al. (2013) who recommend ‘adaptive monitoring’, that is, changing the monitoring scheme through time.

Lastly, we emphasize an observation which might not be intuitive for non-geneticists such as most policy makers: genetic loss may sometimes be slight even if a population is declining, while substantial genetic loss can occur quickly in populations of small, stable size (e.g., *N*_e_ = 50 or 20). This is analogous to species disappearance following habitat loss, which may be small at initial levels of habitat loss, but then accelerates, with potential for further species loss even after habitat loss ceases (Krauss et al. 2010). Similarly, genetic diversity loss associated with population decline occurs not only during the decline itself but also (with increasing rapidity) thereafter in small constant-size populations. As such, stable indicators under population monitoring may mislead if used as a proxy for genetic changes. Similarly, genetic monitoring indicators may mislead if used as a proxy for population size change. A combination of indicators bases on direct population monitoring and genetic diversity is desirable. These results emphasize the importance of monitoring all levels of biodiversity, as genetic biodiversity components may be eroding even while species or population-level indicators show stability.

## Data archiving statement

Simulation files and representative data sets are available from the Dryad Digital Repository: http://doi.org/10.5061/dryad.2m397.

## Supporting Information

Figure S1 Approach to genetic equilibrium over 10 000 simulated generations.

Figure S2 Temporal arrangement of sampling for 20 schemes tested, for weak (90%), moderate (97.5%), and strong (99%) declines.

Figure S3 Pairwise comparison for scenarios from original *N* = 2000 and *N* = 10 000, for number of alleles (*K*).

Figure S4 Pairwise comparison for microsatellites and SNPs, as well as temporal trend in indicator values.

Figure S5 Genetic response to a recovery after 10 generations of reduced population size (reduction from *N* = 2000 to *N* = 50); at right, power of two indicators to detect significant change over time (see also Fig. 3).

Table S1 Carrying capacity and census size of populations during simulations.

Table S2 Complete list of simulated scenarios.

Data S1 Spreadsheet containing complete anova and *t*-test results.
