# Supplementary figures and images for: Comparative evaluation of potential indicators and temporal sampling protocols for monitoring genetic erosion
Source: Evol Appl. 2014 Aug 15;7(9):984–98. doi: 10.1111/eva.12197 (PMC4231590; doi:10.1111/eva.12197)

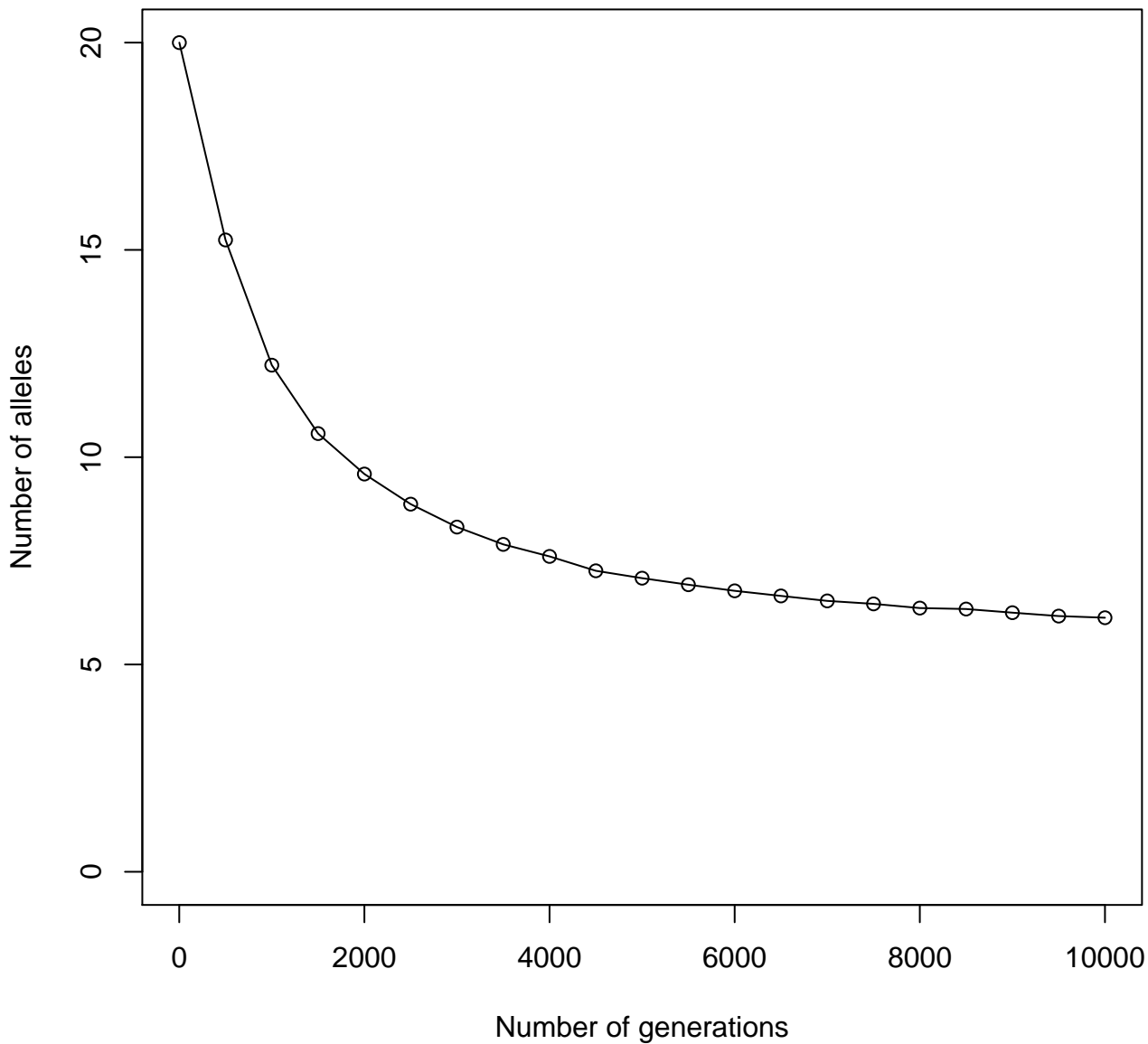

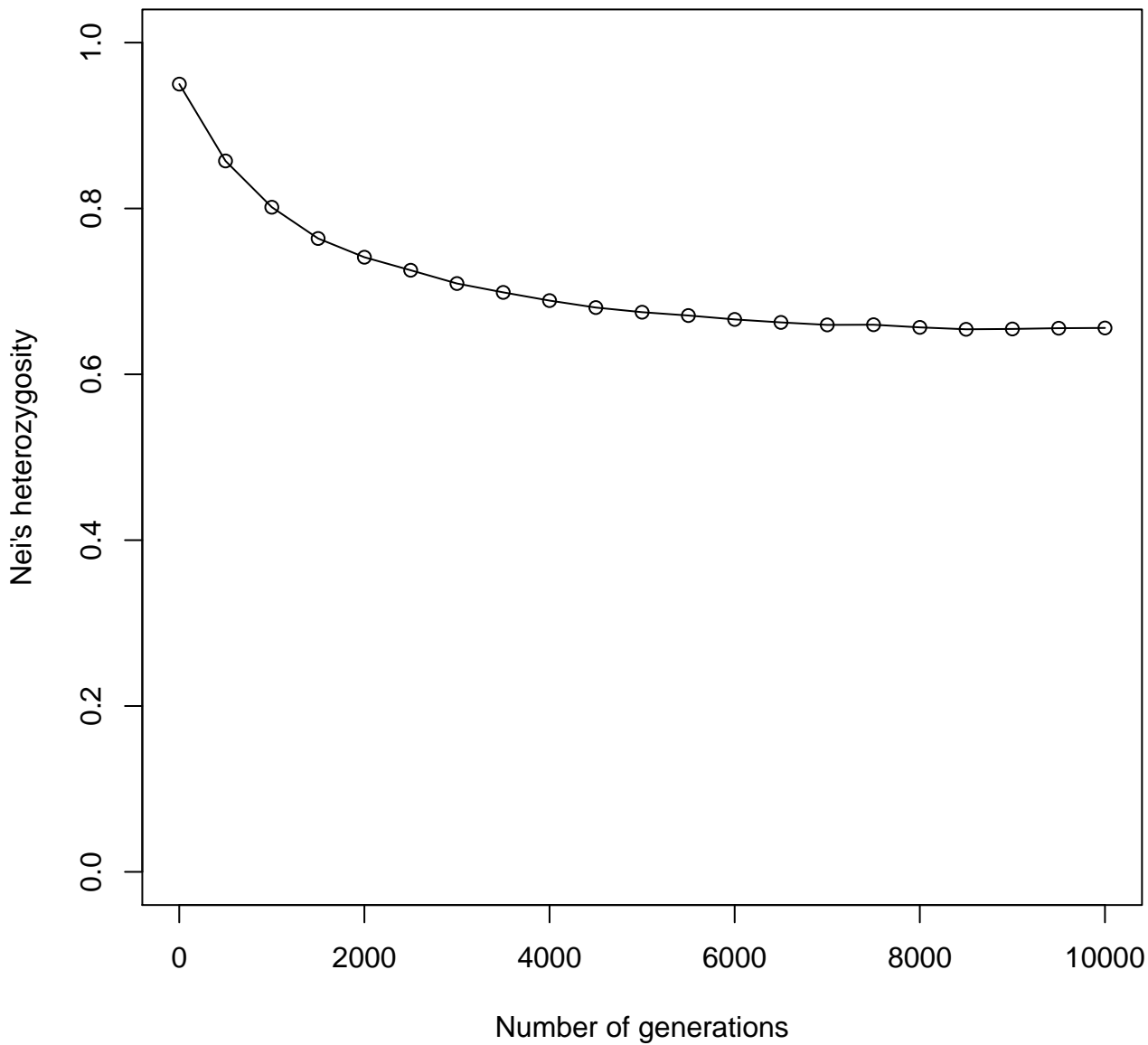

Supplement: Figure S1 — Approach to genetic equilibrium over 10 000 simulated generations. [file eva0007-0984-sd1.pdf]

## Instant from N=2000

90%, K

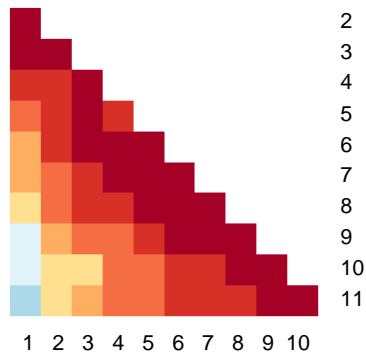

97%, K

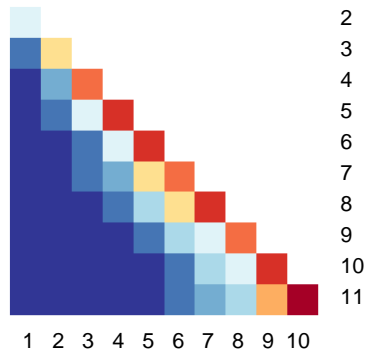

99%, K

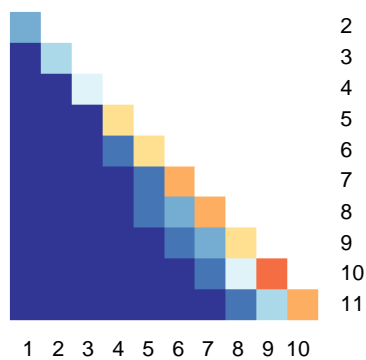

## Instant from N=10000

97%, K

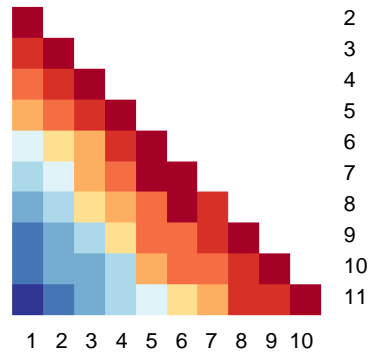

99%, K

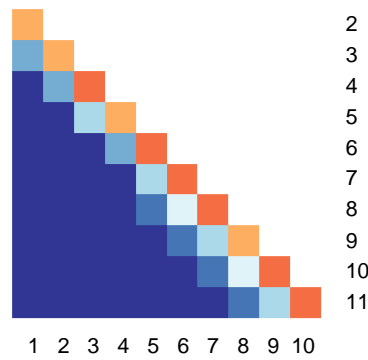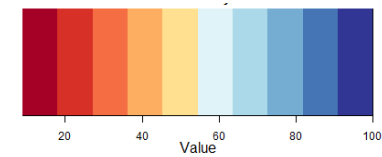

99.5%, K

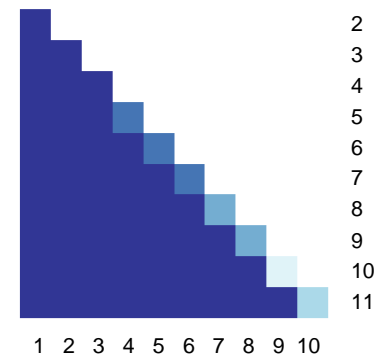

Supplement: Figure S3 — Pairwise comparison for scenarios from original N = 2000 and N = 10 000, for number of alleles (K). [file eva0007-0984-sd3.pdf]
