# Supplementary material for: Comparative evaluation of potential indicators and temporal sampling protocols for monitoring genetic erosion
Source: Evol Appl. 2014 Aug 15;7(9):984–98. doi: 10.1111/eva.12197 (PMC4231590; doi:10.1111/eva.12197)
Supplement: Figure S2 — Temporal arrangement of sampling for 20 schemes tested, for weak (90%), moderate (97.5%), and strong (99%) declines. [file eva0007-0984-sd2.pdf]

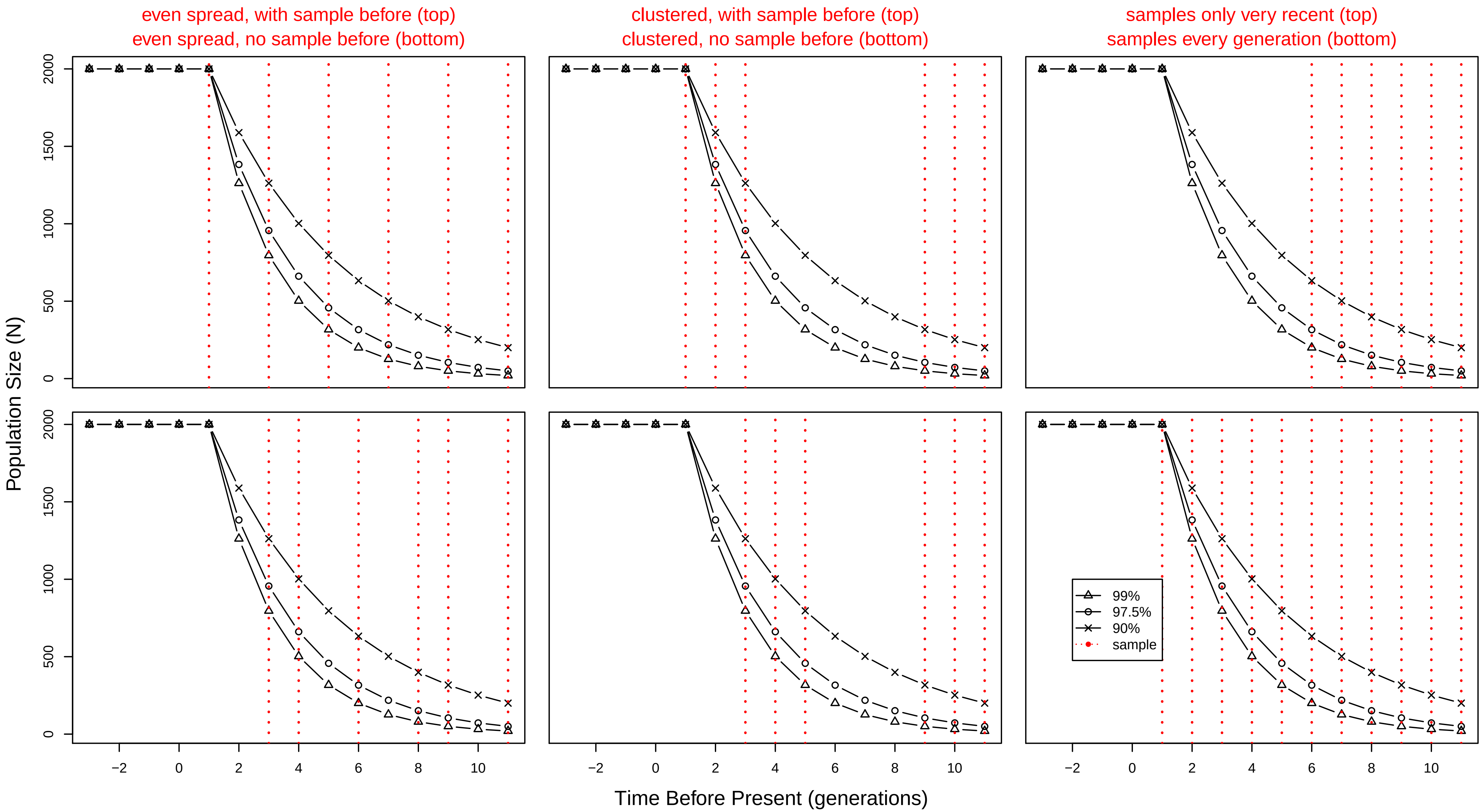

even spread, with sample before (top)
even spread, no sample before (bottom)
clustered, with sample before (top)
clustered, no sample before (bottom)
samples only very recent (top)
samples every generation (bottom)
Population Size (N)
Time Before Present (generations)
99%
97.5%
90%
sample

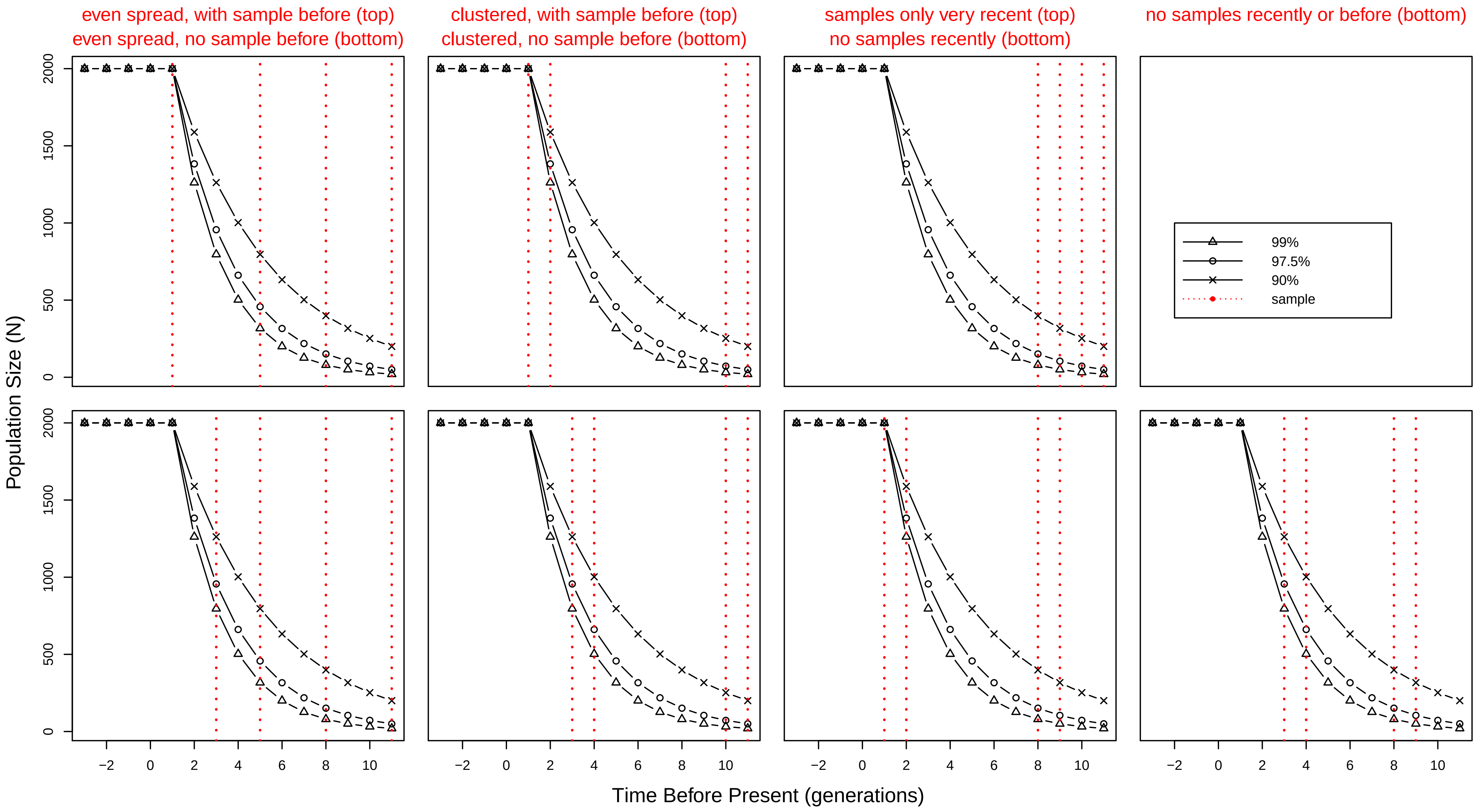

even spread, with sample before (top)
even spread, no sample before (bottom)
clustered, with sample before (top)
clustered, no sample before (bottom)
samples only very recent (top)
no samples recently (bottom)
no samples recently or before (bottom)
99%
97.5%
90%
sample
Population Size (N)
Time Before Present (generations)

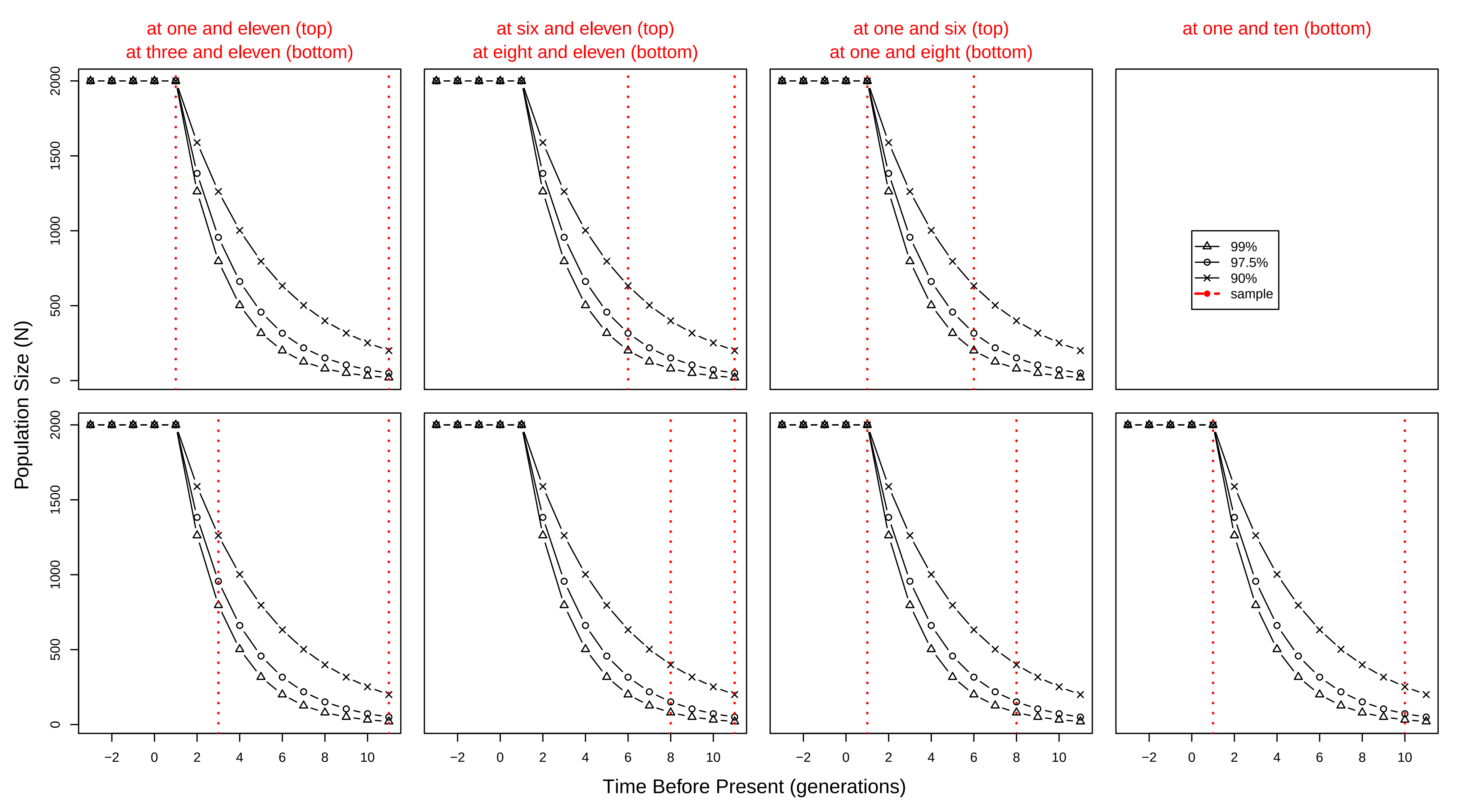

at one and eleven (top)
at three and eleven (bottom)
at six and eleven (top)
at eight and eleven (bottom)
at one and six (top)
at one and eight (bottom)
at one and ten (bottom)
99%
97.5%
90%
sample
Population Size (N)
Time Before Present (generations)

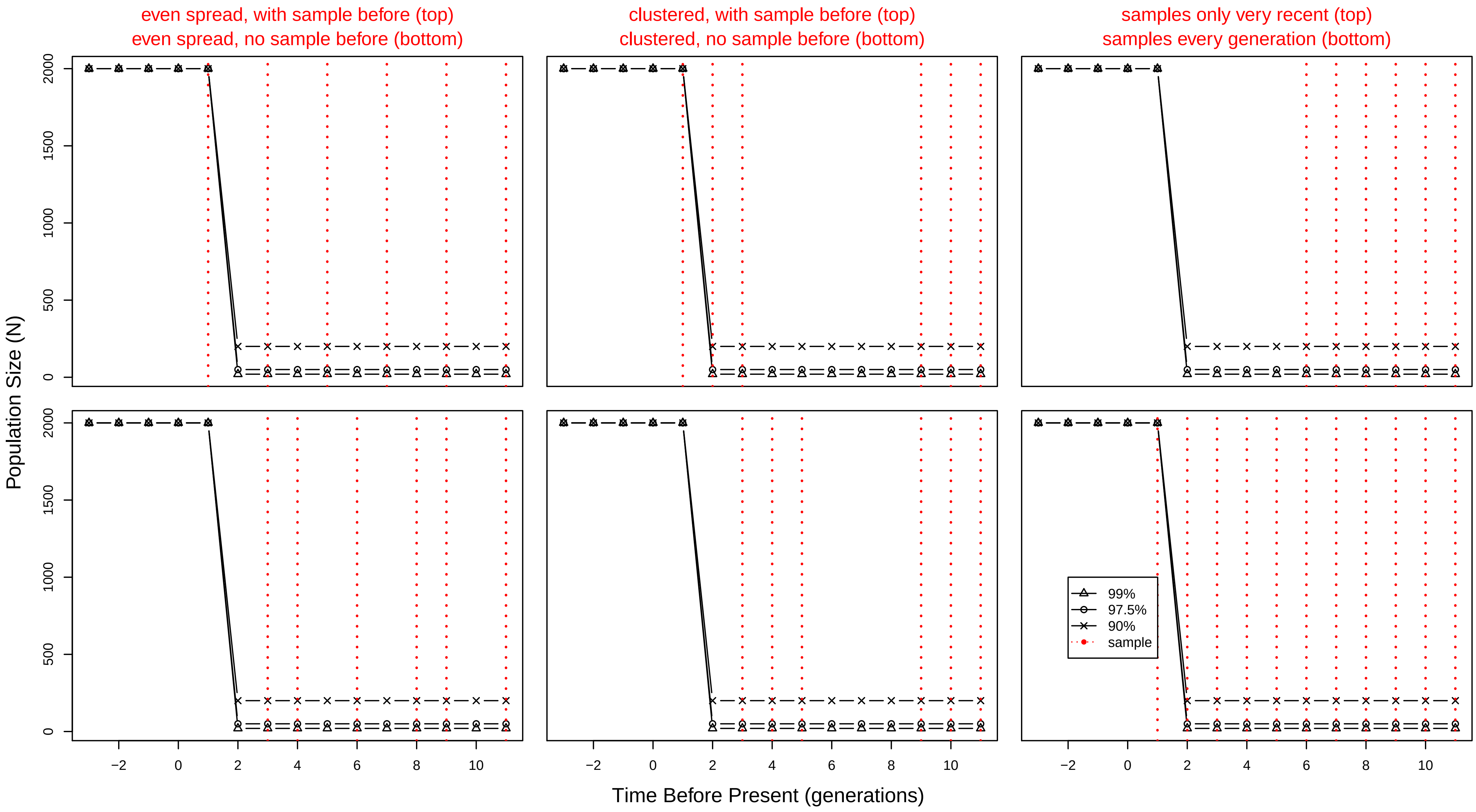
even spread, with sample before (top)
even spread, no sample before (bottom)
clustered, with sample before (top)
clustered, no sample before (bottom)
samples only very recent (top)
samples every generation (bottom)
Population Size (N)
Time Before Present (generations)
0
500
1000
1500
2000
−2
0
2
4
6
8
10
99%
97.5%
90%
sample

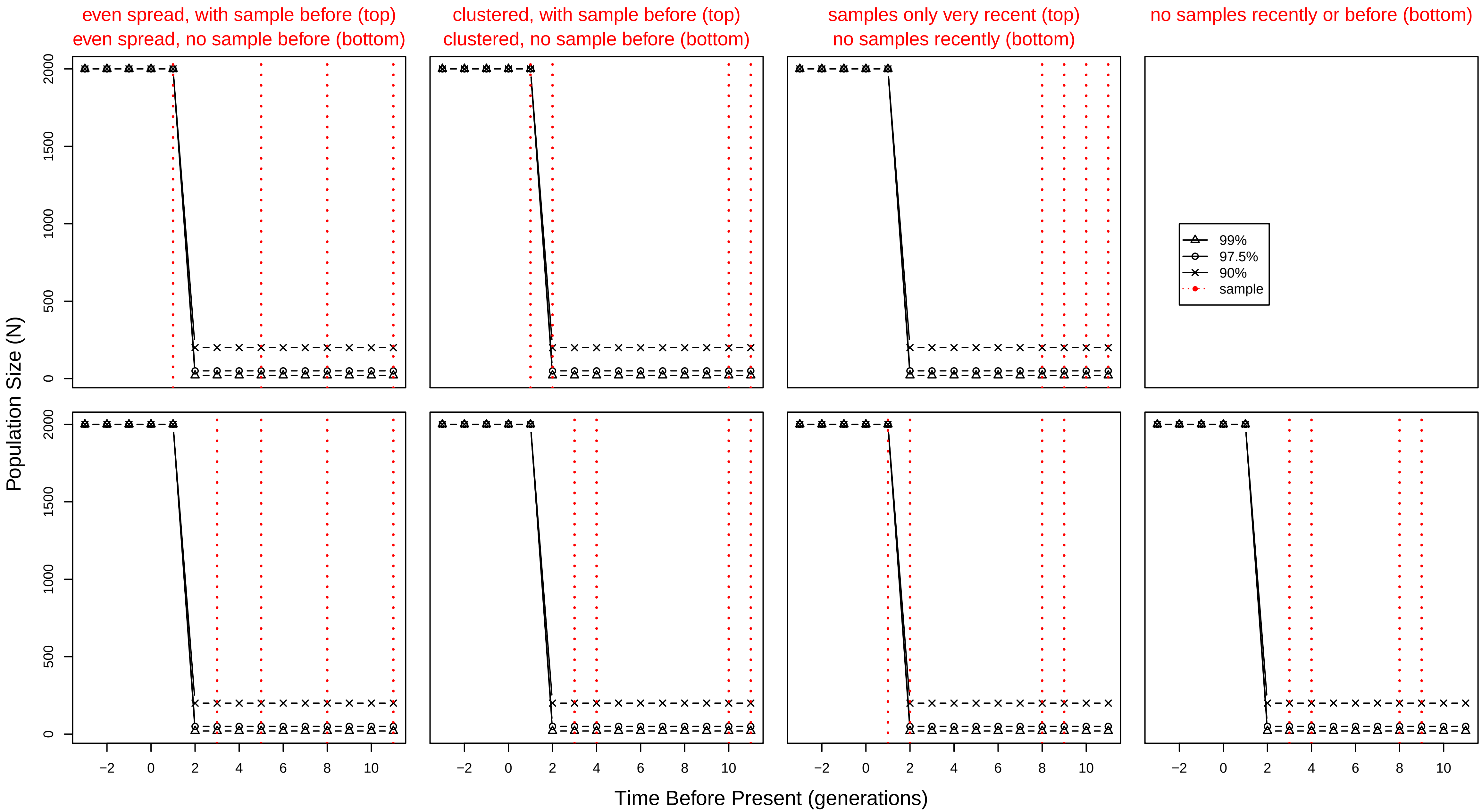
even spread, with sample before (top)
even spread, no sample before (bottom)
clustered, with sample before (top)
clustered, no sample before (bottom)
samples only very recent (top)
no samples recently (bottom)
no samples recently or before (bottom)
99%
97.5%
90%
sample
Population Size (N)
Time Before Present (generations)

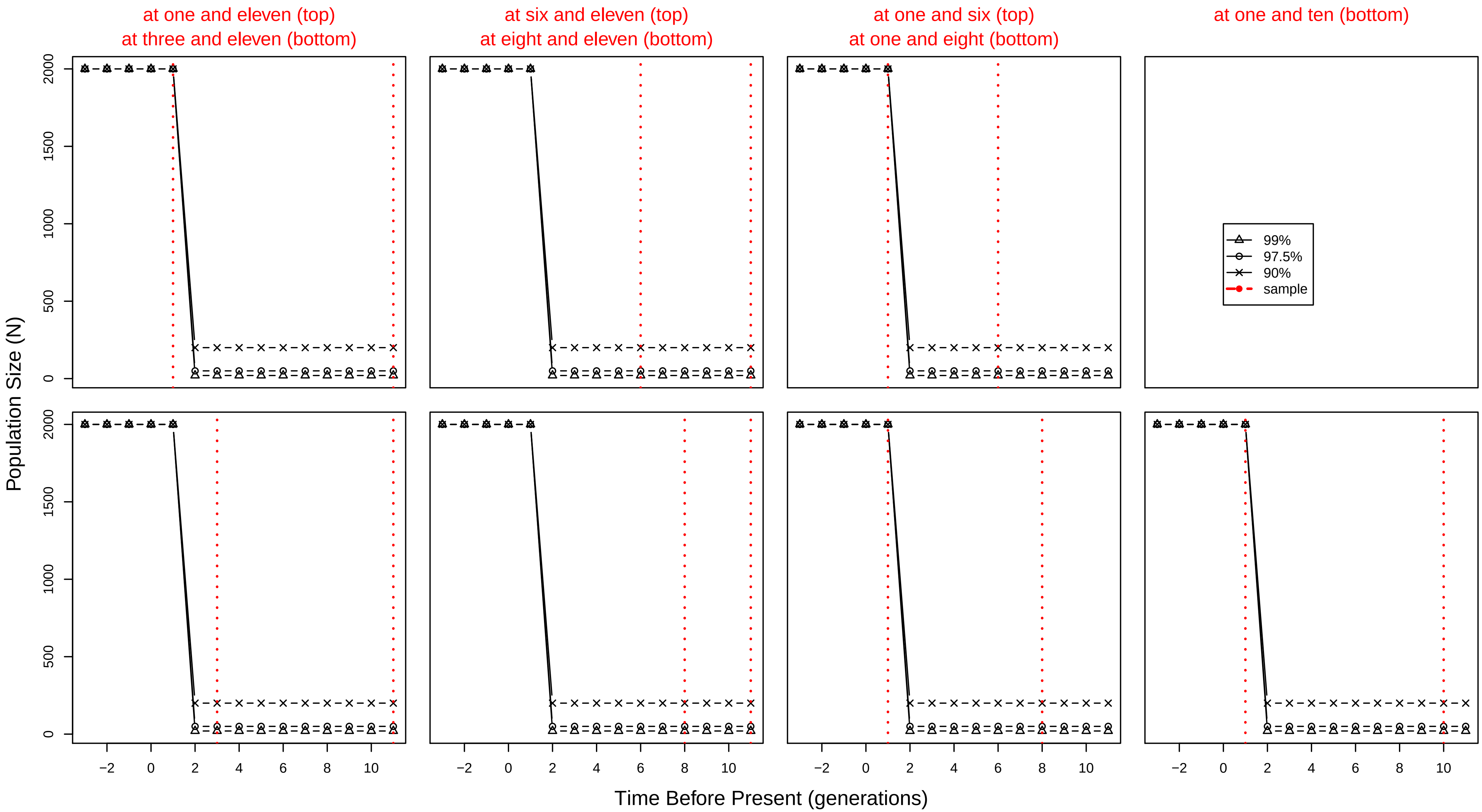
at one and eleven (top)
at three and eleven (bottom)
at six and eleven (top)
at eight and eleven (bottom)
at one and six (top)
at one and eight (bottom)
at one and ten (bottom)
99%
97.5%
90%
sample
Population Size (N)
Time Before Present (generations)
