# Supplementary material for: Comparative evaluation of potential indicators and temporal sampling protocols for monitoring genetic erosion
Source: Evol Appl. 2014 Aug 15;7(9):984–98. doi: 10.1111/eva.12197 (PMC4231590; doi:10.1111/eva.12197)
Supplement: Figure S4 — Pairwise comparison for microsatellites and SNPs, as well as temporal trend in indicator values. [file eva0007-0984-sd4.pdf]

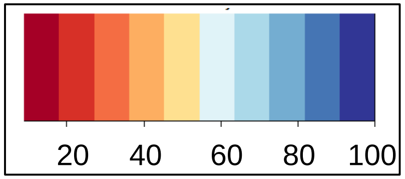

2500 SNPs, exponential

97%, K

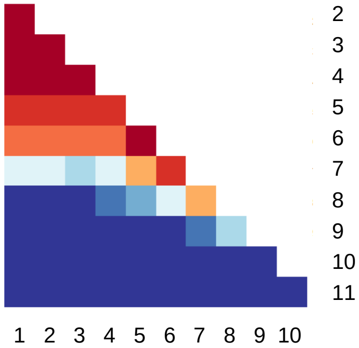

97%, He

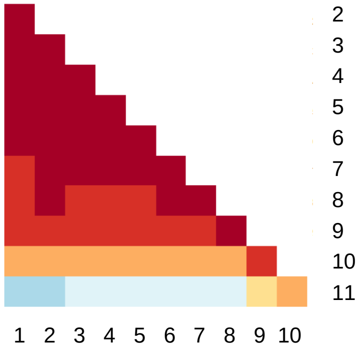

97%, Ho

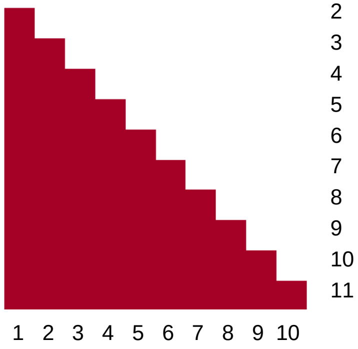

250 microsats, exponential

97%, K

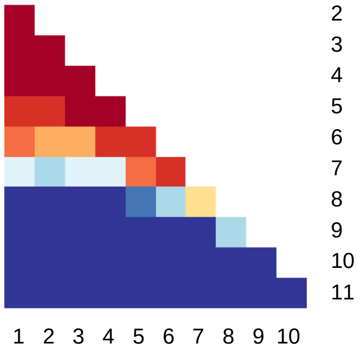

97%, He

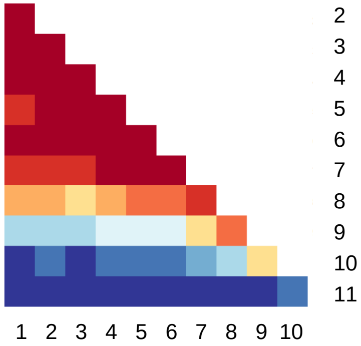

97%, Ho

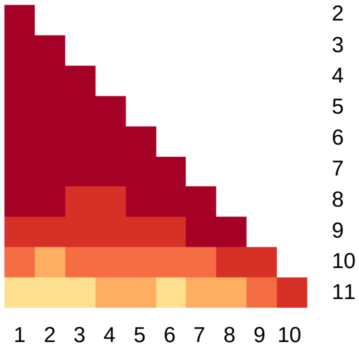

More on next 2 pages

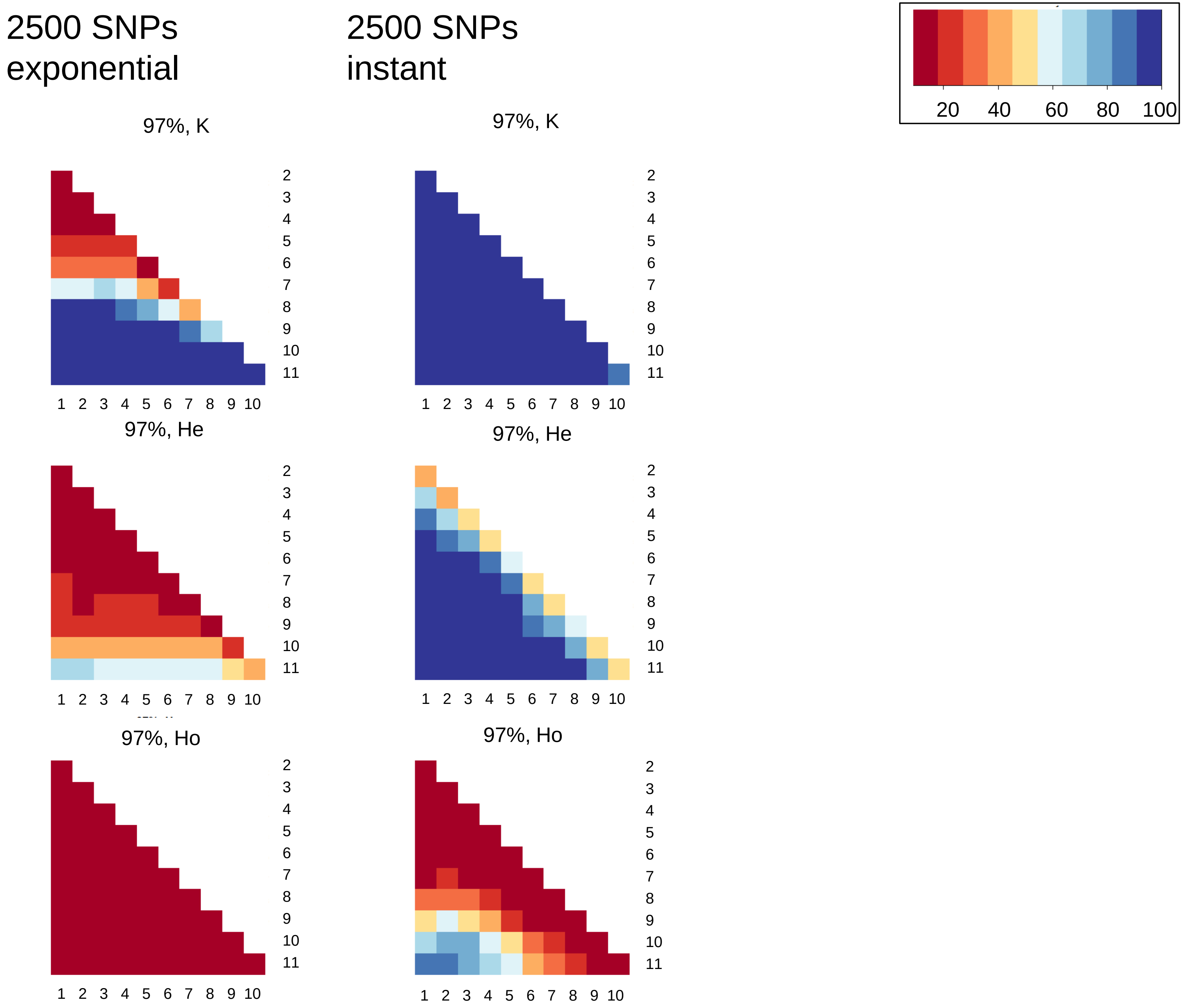
2500 SNPs
exponential
2500 SNPs
instant
20
40
60
80
100
97%, K
97%, He
97%, Ho
1 2 3 4 5 6 7 8 9 10
2 3 4 5 6 7 8 9 10 11

2500 SNPs exponential

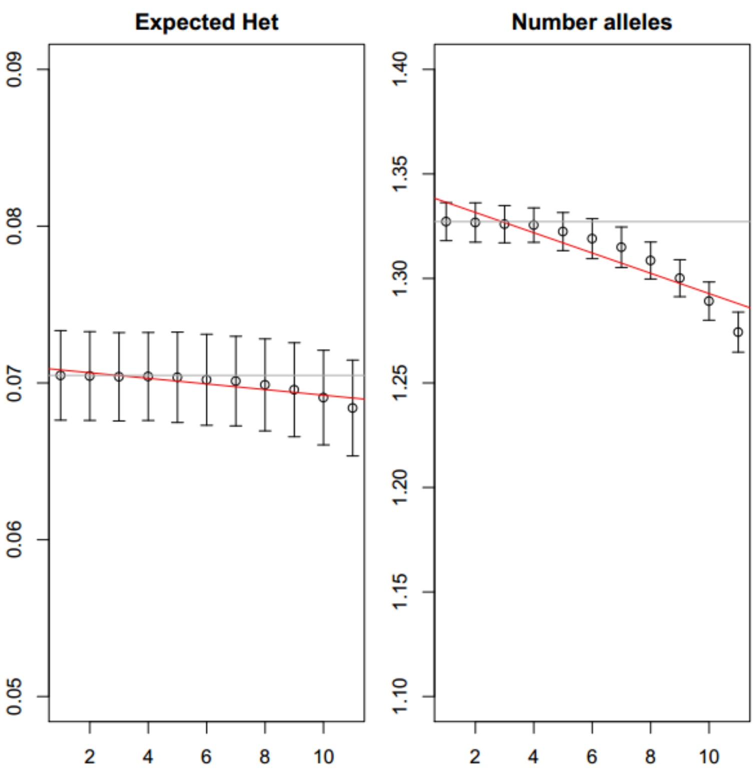

2500 SNPs instant

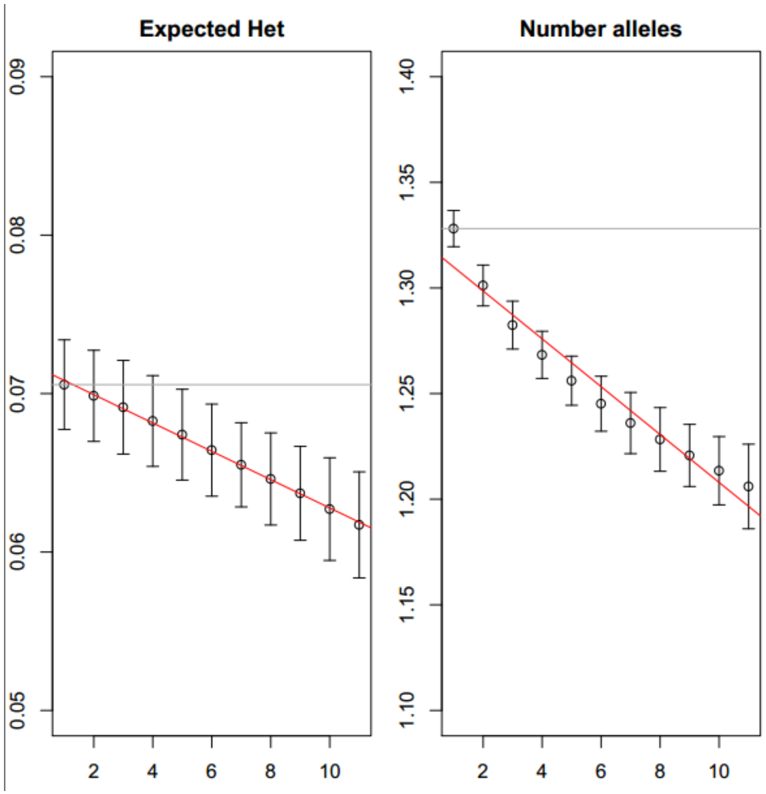

Generation number
