# Supplementary material for: Comparative evaluation of potential indicators and temporal sampling protocols for monitoring genetic erosion
Source: Evol Appl. 2014 Aug 15;7(9):984–98. doi: 10.1111/eva.12197 (PMC4231590; doi:10.1111/eva.12197)
Supplement: Table S2 — Complete list of simulated scenarios. [file eva0007-0984-sd7.doc]

**Supplemental Table 2:** Situations simulated for testing indicators

| **Population change** | **Type change** | **Percentage of decline** | **Number loci (microsatellites unless noted)** | **Sample size (number of individuals)** |
| --- | --- | --- | --- | --- |
| 2000 to 200 | exponential | 90 | 20 | 50 |
| 2000 to 50 | exponential | 97.5 | 20 | 50 |
| 2000 to 20 | exponential | 99 | 20 | 50* |
| 2000 to 200 | instant | 90 | 20 | 50 |
| 2000 to 50 | instant | 97.5 | 20 | 50 |
| 2000 to 20 | instant | 99 | 20 | 50* |
| 2000 to 200 | exponential | 90 | 250 | 50 |
| 2000 to 50 | exponential | 97.5 | 250 | 50 |
| 2000 to 20 | exponential | 99 | 250 | 50* |
| 2000 to 200 | exponential | 90 | 20 | Whole population |
| 2000 to 50 | exponential | 97.5 | 20 | Whole population |
| 2000 to 20 | exponential | 99 | 20 | Whole population |
| 10000 to 300 | instant | 97 | 20 | 50 |
| 10000 to 100 | instant | 99 | 20 | 50 |
| 10000 to 50 | instant | 99.5 | 20 | 50 |
| 2000 to 50 to 2000 (2 generations at 50) | instant plus recovery | 97.5 | 20 | 50 |
| 2000 to 50 to 2000 (10 generations at 50) | instant plus recovery | 97.5 | 20 | 50 |
| 2000 to 50 to 2000 (20 generations at 50) | instant plus recovery | 97.5 | 20 | 50 |
| 2000 to 50 | exponential | 97.5 | 2500 SNPs | 50 |
| 2000 to 50 | instant | 97.5 | 2500 SNPs | 50 |

* Sample size was smaller for the latest generations, when total population size was below 50.
