# Supplementary material for: Comparative evaluation of potential indicators and temporal sampling protocols for monitoring genetic erosion
Source: Evol Appl. 2014 Aug 15;7(9):984–98. doi: 10.1111/eva.12197 (PMC4231590; doi:10.1111/eva.12197)
Supplement: Data S1 — Spreadsheet containing complete anova and t-test results. [file eva0007-0984-sd8.xlsx]

**Supplemental Table 1:** Carrying capacity (K) compared to actual mean population size (N) considering stochastic processes in the simulator Nemo, averaged over 100 replicates

| type | Exponential | | | | | | Instant | | | | | |
| --- | --- | --- | --- | --- | --- | --- | --- | --- | --- | --- | --- | --- |
| % | 90 | | 97.5 | | 99 | | 90 | | 97.5 | | 99 | |
| T | N | K | N | K | N | K | N | K | N | K | N | K |
| 1 | 1995 | 2000 | 1995 | 2000 | 1995 | 2000 | 1995 | 2000 | 1995 | 2000 | 1995 | 2000 |
| 2 | 1588 | 1589 | 1382 | 1383 | 1260 | 1262 | 200 | 200 | 50 | 50 | 20 | 20 |
| 3 | 1262 | 1262 | 956 | 956 | 796 | 796 | 195.1 | 200 | 46.7 | 50 | 17.81 | 20 |
| 4 | 1002 | 1002 | 660 | 661 | 502 | 502 | 193.1 | 200 | 44.17 | 50 | 16.45 | 20 |
| 5 | 796 | 796 | 456 | 457 | 316 | 317 | 189.3 | 200 | 43.12 | 50 | 15.49 | 20 |
| 6 | 632 | 632 | 316 | 316 | 200 | 200 | 190 | 200 | 42.79 | 50 | 14.04 | 20 |
| 7 | 502 | 502 | 218 | 219 | 126 | 126 | 186.3 | 200 | 41.82 | 50 | 14.32 | 20 |
| 8 | 398 | 399 | 150 | 151 | 78 | 80 | 186.2 | 200 | 40.24 | 50 | 13.31 | 20 |
| 9 | 316 | 317 | 104 | 105 | 49.97 | 50 | 184.7 | 200 | 39.43 | 50 | 13.59 | 20 |
| 10 | 252 | 252 | 71.96 | 72 | 29.93 | 32 | 181.7 | 200 | 38.58 | 50 | 12.96 | 20 |
| 11 | 200 | 200 | 49.89 | 50 | 19.68 | 20 | 183.4 | 200 | 37.17 | 50 | 12.37 | 20 |

Note that the actual population size is typically slightly less than the carrying capacity, due to demographic stochasticity in the individual-based simulations. This is only noticeably smaller for the instant declines, and only substantially so for the instant 99% decline, in which the mean N might be 12 by the final generation, which is only 60% of the carrying capacity (K=20). This would likely make the test more powerful because the population (at size N) will lose diversity faster than suggested by the carrying capacity (size K). Thus the results in this document may be slightly liberal estimates of power in this particular situation.
