# Supplementary material for: Comparative evaluation of potential indicators and temporal sampling protocols for monitoring genetic erosion
Source: Evol Appl. 2014 Aug 15;7(9):984–98. doi: 10.1111/eva.12197 (PMC4231590; doi:10.1111/eva.12197)
Supplement: Supplementary file 9 [file eva0007-0984-sd9.docx]

**Supplemental Materials**

Supplemental Figure 1: Approach to genetic equilibrium over 10,000 simulated generations


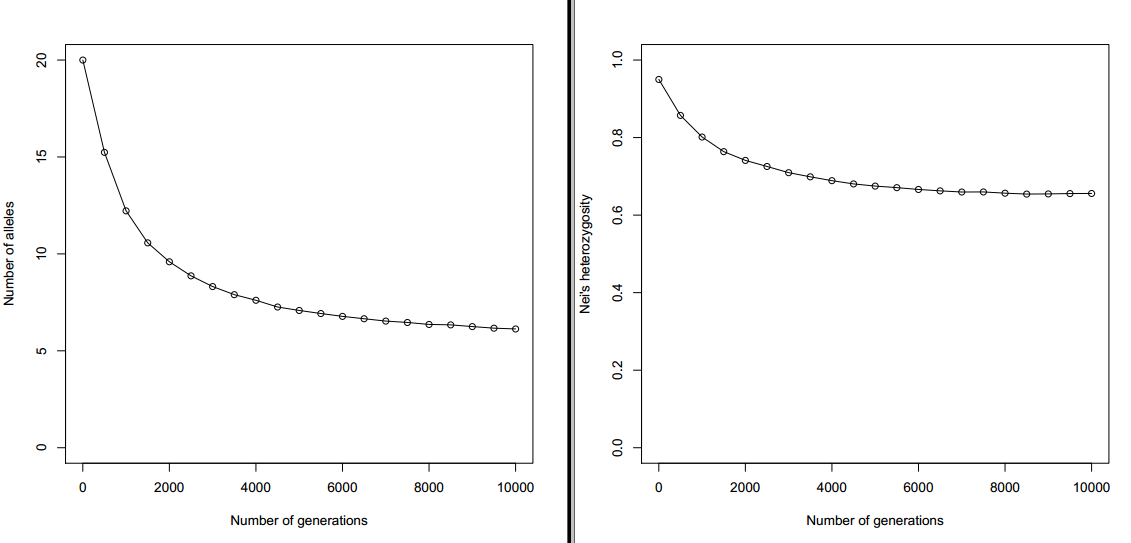


Supplemental Figure 2: Temporal arrangement of sampling for 20 schemes tested, for weak (90%), moderate (97.5%), and strong (99%) declines. Sampling events are indicated by stippled vertical lines.

Supplemental Figure 3: Pairwise comparison for scenarios from original N=2000 and N=10000, for number of alleles (K)

Supplemental Figure 4: Pairwise comparison for microsatellites and SNPs, as well as temporal trend in indicator values.


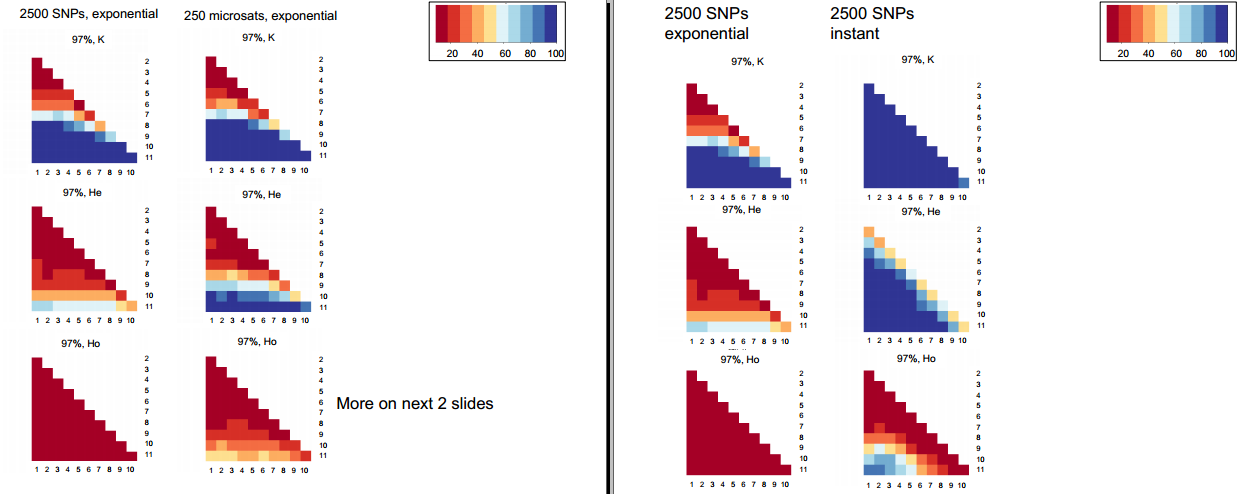


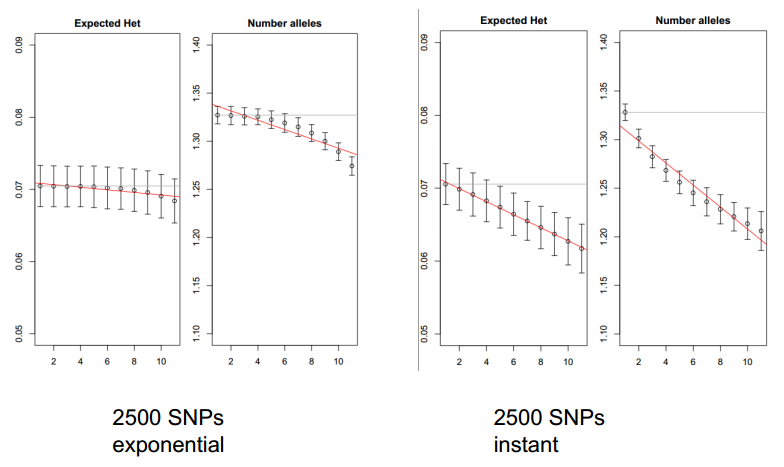


Supplemental Figure 5: Genetic response to a recovery after ten generations of reduced population size (reduction from N=2000 to N=50); at right, power of two indicators to detect significant change over time (see also Figure 3)

Supplemental Table 1: Carrying capacity and census size of populations during simulations

Supplemental Table 2: Complete list of simulated scenarios

Supplemental Spreadsheet: Complete ANOVA and t-test results
