# Supplementary material for: Comparative evaluation of potential indicators and temporal sampling protocols for monitoring genetic erosion
Source: Evol Appl. 2014 Aug 15;7(9):984–98. doi: 10.1111/eva.12197 (PMC4231590; doi:10.1111/eva.12197)
Supplement: Figure S5 — Genetic response to a recovery after 10 generations of reduced population size (reduction from N = 2000 to N = 50); at right, power of two indicators to detect significant change over time (see also Fig. 3). [file eva0007-0984-sd5.pdf]

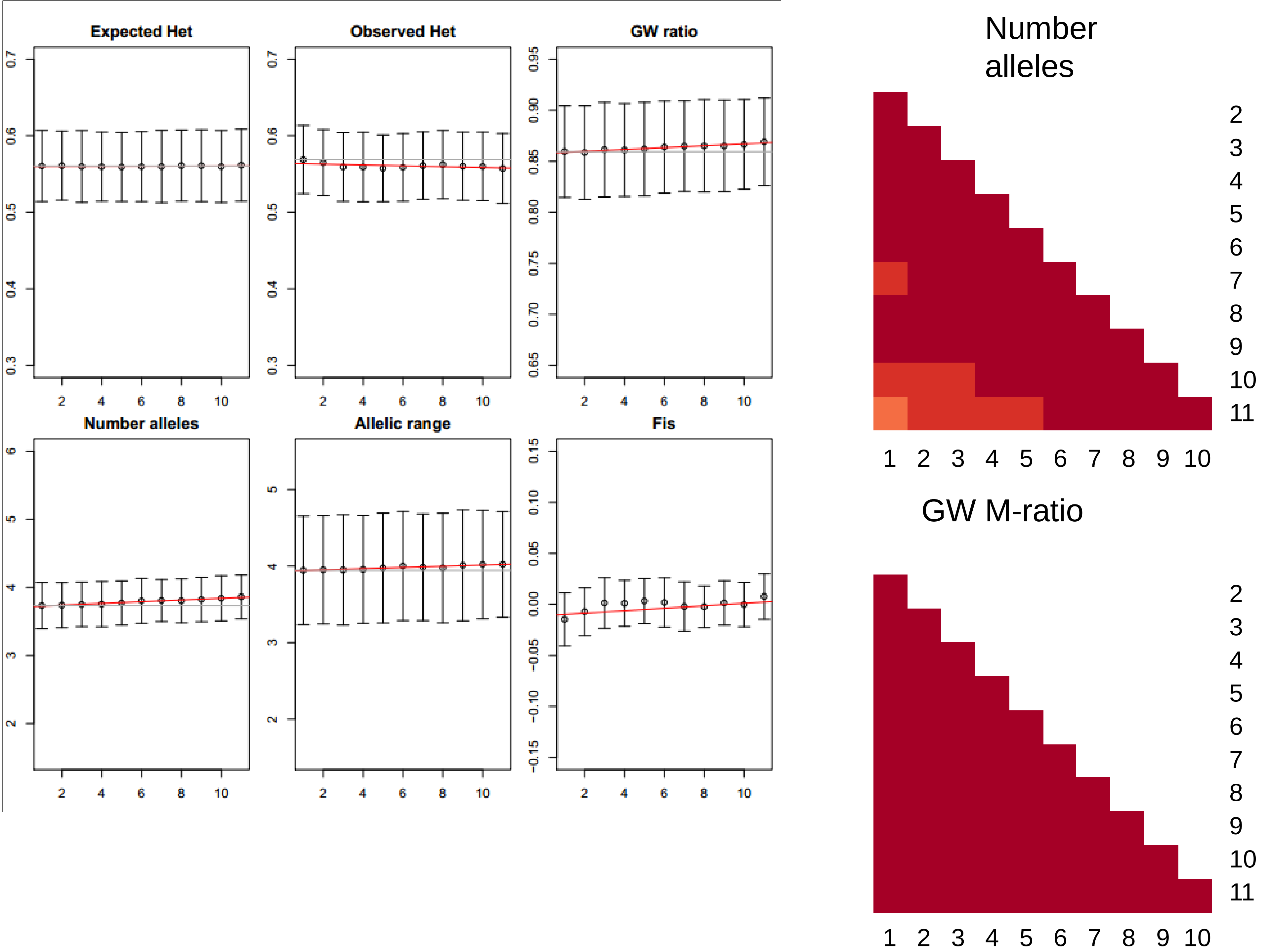

Expected Het
Observed Het
GW ratio
Number alleles
Allelic range
Fis
Number alleles
GW M-ratio
